# Autophagy activity contributes to the impairment of social recognition in *Epac2*^−/−^ mice

**DOI:** 10.1186/s13041-021-00814-6

**Published:** 2021-06-28

**Authors:** Ji-Hye Kwak, You-kyung Lee, Mi-Hee Jun, Mootaek Roh, Hyunhyo Seo, Juhyun Lee, Kyungmin Lee, Jin-A Lee

**Affiliations:** 1 Behavioral Neural Circuitry and Physiology Laboratory, Department of Anatomy, Brain Science and Engineering Institute, School of Medicine, Kyungpook National University, Daegu, 41944 South Korea; 2 Department of Biological Sciences and Biotechnology, College of Life Sciences and Nanotechnology, Hannam University, Daejeon, South Korea

**Keywords:** Autophagy, Epac2, Social recognition, Neurodevelopmental disorders

## Abstract

Autophagy is a lysosomal degradation pathway that regulates cellular homeostasis. It is constitutively active in neurons and controls the essential steps of neuronal development, leading to its dysfunction in neurodevelopmental disorders. Although mTOR-associated impaired autophagy has previously been reported in neurodevelopmental disorders, there is lack of information about the dysregulation of mTOR-independent autophagy in neurodevelopmental disorders. In this study, we investigated whether the loss of *Epac2,* involved in the mTOR-independent pathway, affects autophagy activity and whether the activity of autophagy is associated with social–behavioral phenotypes in mice with *Epac2* deficiencies*.* We observed an accumulation of autophagosomes and a significant increase in autophagic flux in Epac2-deficient neurons, which had no effect on mTOR activity. Next, we examined whether an increase in autophagic activity contributed to the social behavior exhibited in *Epac2*^*−/−*^ mice. The social recognition deficit observed in *Epac2*^*−/−*^ mice recovered in double transgenic *Epac2*^*−/−*^: *Atg5*^+*/−*^ mice. Our study suggests that excessive autophagy due to Epac2 deficiencies may contribute to social recognition defects through an mTOR-independent pathway.

**Supplementary Information:**

The online version contains supplementary material available at 10.1186/s13041-021-00814-6.

Macroautophagy (hereafter autophagy) is a dynamic cellular pathway that regulates the lysosomal degradation of cytosolic components, including organelles, proteins, lipids, DNA, RNA, or unwanted materials within cells [1]. Autophagy is a tightly regulated process, conducted by several autophagy-related (ATG) proteins in neurons. Knockout of key ATG components, like ATG5 or ATG7, causes accumulation of ubiquitinated proteins and neurodegeneration, suggesting its importance in neuronal health [2, 3]. Regarding signaling pathways, autophagy is regulated by mTOR (mammalian target of rapamycin), which senses and integrates several intracellular and environmental cues to orchestrate major processes, including cell growth and metabolism, or mTOR-independent pathways, like cAMP (3′–5′-cyclic adenosine monophosphate), Ca^2+^, or IP_3_ (Inositolphosphoinositide-3) [4]. Thus far, most reports indicate that mTOR pathway dysregulation, which regulates neurodevelopment or synaptic plasticity, is linked to impaired autophagy, leading to mTOR-associated brain diseases, including autism spectrum disorders (ASD) [5, 6]. However, to better understand the role of autophagy in neurodevelopment, synaptic function, or neurological disorders, it is also important to investigate mTOR-independent autophagy in brain function using in vitro and in vivo models.

Previous investigations have reported that elevated intracellular cAMP inhibits autophagy and is mediated by exchange protein activated by cAMP (Epac) [7, 8]. Epac2 is highly expressed in the brain and is an upstream activator of the small GTPase Ras family [9]. Several animal studies have identified behavioral phenotypes in *Epac2* knockout (*Epac2*^*−/−*^) mice consistent with the link to ASD susceptibility [10], including impaired memory, behavioral inflexibility, and altered social interactions [11, 12]. However, whether the loss of *Epac2* affects autophagy activity and whether autophagy is associated with social–behavioral phenotypes in *Epac2*^*−/−*^ mice remains unclear. Therefore, we investigated autophagy in *Epac2*^*−/−*^ mice to assess whether it affects the social–behavioral phenotype observed in these models.

First, we investigated the involvement of autophagy in *Epac2*^*−/−*^ mice by transfecting HyD-LIR-based autophagosome sensors (HyD-LIR-GFP), which could detect endogenous LC3 or GABARAP family proteins in autophagosomes, into cultured cortical neurons (div1) [13]. Two days after transfection, the number of HyD-LIR-GFP-positive autophagosomes in Epac2 deficient neurons was significantly accumulated compared with that of the wild-type cortical neurons, in the presence of a lysosomal inhibitor (chloroquine, CQ), indicating that autophagic activity was higher in Epac2-deficient neurons than in wild-type neurons (Fig. 1A, B).

**Fig. 1:**
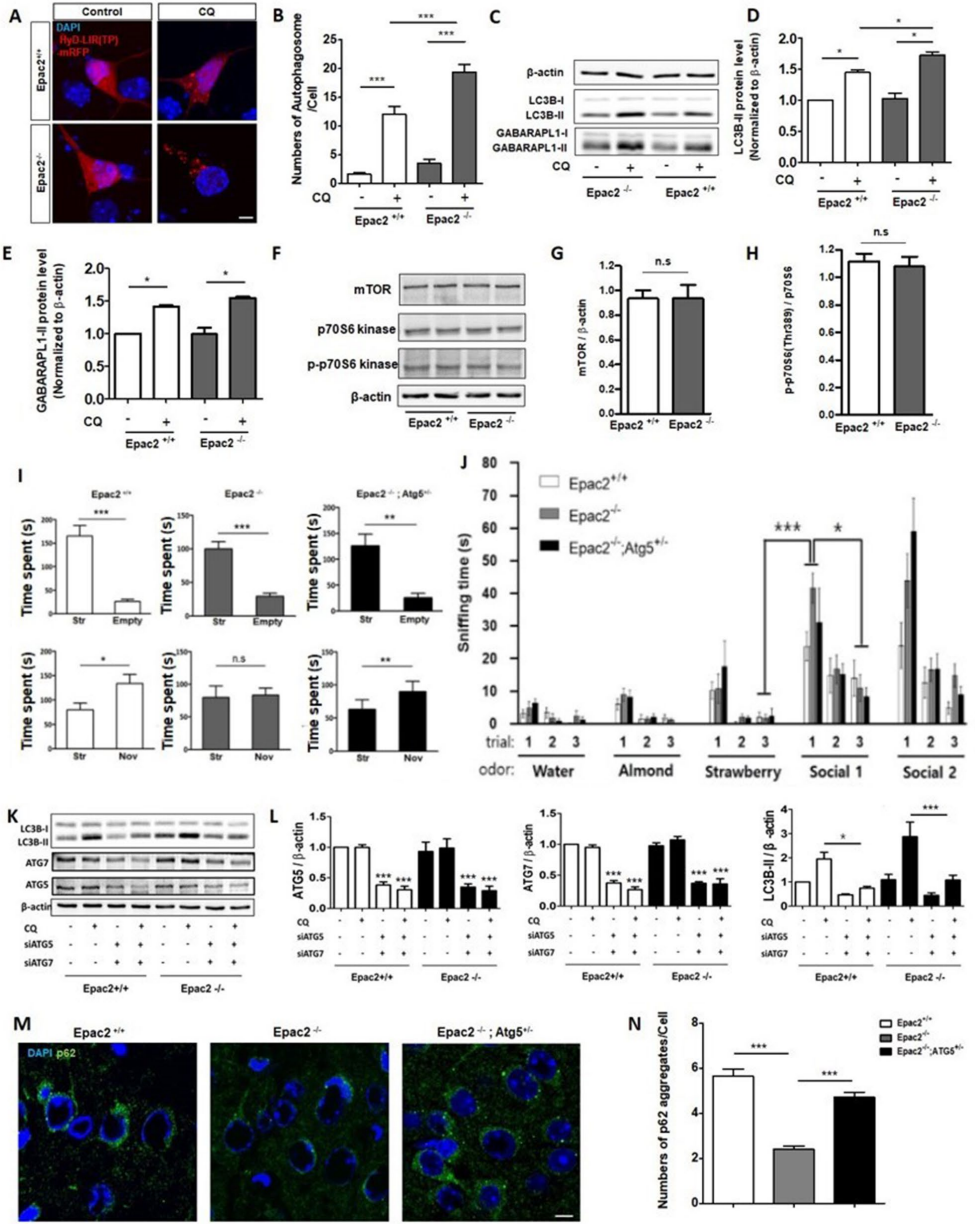
The Excessive autophagic activity contributes to autistic behavior in *Epac2*^*−/−*^ mice. **A** Representative confocal images demonstrate HyD-LIR-GFP-positive autophagosome in wild-type cortical and *Epac2*^*−/−*^ neurons, in the presence or absence of chloroquine (CQ; 50 μM), for 24 h. Scale bar, 10 μm. **B** Bar graph represents the number of HyD-LIR-GFP-positive autophagosomes in wild-type cortical and *Epac2*^*−/−*^ neurons. **C** *Epac2*^+*/*+^ or *Epac2*^*−/−*^ neurons were incubated in the presence or absence of CQ. Then, the cell lysates were subjected to Western blotting with anti-LC3B, anti-GABARAPL1, or anti-β-actin antibodies. **D**, **E** The LC3-II and GABARAPL1-II levels were normalized similarly to that of β-actin. Western blot (**F**) and (**G**, **H**) quantitative analysis indicating the protein levels of mTOR, p70S6 kinase, and phosphorylated p70S6 kinase (p-p70S6 kinase) in the cultured cortical neurons of *Epac2*^+*/*+^ and *Epac2*^*−/−*^ mice (n.s, no significance). **I** All *Epac2*^+*/*+^ and *Epac2*^*−/−*^ mice showed a significant preference for exploring a stranger mouse rather than an empty cage. Contrary to *Epac2*^+*/*+^, *Epac2*^*−/−*^ mice exhibited no difference in durations exploring a stranger mouse *vs.* a familiar one. *Epac2*^*−/−*^*:Atg5*^+*/−*^ mice showed sociability and preference for social novelty similar to *Epac2*^+*/*+^ mice, suggesting the rescue of deficit in social novelty recognition of *Epac2*^*−/−*^ mice (n.s, no significance; Str, stranger mouse; Nov, novel mouse). **J** *Epac2*^*−/−*^ mice can detect and discriminate nonsocial and social olfactory cues with normal dishabituation to novel social odor and habituation to repeated same social odor. **K**, **M** Western blotting and quantitative analysis indicating the protein levels of ATG5, ATG7, LC3B, and β-actin in cultured cortical neurons in *Epac2*^+*/*+^ and *Epac2*^*−/−*^ mice in the presence or absence of siRNAs against ATG5 or ATG7. (M, N) Representative confocal images and quantitative analysis show p62-positive aggregates in wild-type, *Epac2*^*−/−*^*,* and *Epac2*^*−/−*^*:Atg5*^+*/−*^ cortical neurons. Values are presented as a mean ± standard error of the mean (SEM). *p < 0.05, **p < 0.01, ***p < 0.001. Scale bar, 10 μm

To further investigate whether autophagy was upregulated due to Epac2 deficiencies, we performed an autophagic flux assay by Western blot, using anti-LC3B or anti-GABARAPL1 antibodies in the presence or absence of a lysosomal inhibitor in *Epac2*^+*/*+^ or *Epac2*^*−/−*^ cortical neurons. As shown in Fig. 1C–E, the expression levels of LC3-II and GABARAPL1-II were significantly increased with lysosomal inhibition in *Epac2*^*−/−*^ neurons compared with *Epac2*^+*/*+^ neurons. These results indicate that Epac2 deficiencies abnormally upregulated autophagy activity.

Next, we examined whether the mTOR pathway affected abnormal autophagic activity in *Epac2*^*−/−*^ mice. Therefore, the protein levels of mTOR, p70S6 kinase, and phosphorylated p70S6 kinase was evaluated. No significant difference was noted in mTOR levels or activity, as shown in Fig. 1F–H. This data suggests that *Eapc2* gene deficiencies could induce changes in mTOR-independent pathways, leading to abnormally enhanced autophagic activity in the cortical neurons.

Next, we investigated the relationship between autophagy activity and autistic phenotype, including social recognition deficit [14]. A three-chambered social approach test was used to assess the social behaviors of *Epac2*^*−/−*^ mice with hyperactive autophagic activity. We found that *Epac2*^*−/−*^ mice had normal sociability but an abnormal preference for social novelty, suggesting a deficit in social recognition (Fig. 1I). Next, we examined their abilities to detect social olfactory cues using an olfactory habituation/dishabituation task [15]. Both *Epac2*^+*/*+^ and *Epac2*^*−/−*^ mice could detect and discriminate nonsocial and social olfactory cues, with normal dishabituation to novel social odor and habituation to repeated same social odor (Fig. 1J). These data suggest that abnormal preference for social novelty in *Epac2*^*−/−*^ mice is not due to a dysfunction in detecting social odor cues.

Next, to observe whether an abnormal autophagic activity could be associated with abnormal social recognition, we conducted an assay to normalize the abnormal autophagic activity enhanced by *Epac2* deficiencies. Cultured cortical neurons were used as autophagic flux assays, which can be performed using this in vitro system, and found reductions in ATG5 or ATG7, siRNA transfection, and decreased excessive autophagic flux in the cultured cortical neurons of *Epac2*^*−/−*^ mice (Fig. 1K, L). Next, we performed ATG5 knockdown in vivo using *Epac2*^*−/−*^ mice and generated *Epac2*^*−/−*^*:Atg5*^+*/−*^ mice to downregulate abnormally enhanced autophagic activity. We confirmed that within the cortical neurons of *Epac2*^*−/−*^ mice, aggregation of p62 protein was reduced and was restored in the cortical neurons of *Epac2*^*−/−*^*:Atg5*^+*/−*^ mice in vivo (Fig. 1M, N).

Interestingly, normal sociability and preference for social novelty was observed in the *Epac2*^*−/−*^*:Atg5*^+*/−*^ mice, with the normal ability of olfactory discrimination similar to *Epac2*^+*/*+^ mice, suggesting that the deficit in social recognition of *Epac2*^*−/−*^ mice was rescued through crossing with *Atg5*^+*/−*^ mice (Fig. 1I, J). Altogether, our results suggest that Epac2 contributes to the maintenance of basal autophagy activity and normal social recognition as a basis for normal social behavior by suppressing autophagy over activation.

Thus far, impaired or insufficient autophagy has mostly been described in neurological disorders. However, the role of abnormal autophagy upregulation without prominent cell death in brain function or neurological disorders remains unclear [1, 16]. Moreover, the role of mTOR-independent autophagy in brain functioning and the relationship between hyperactive autophagy and social–behavioral defects remain largely unknown. Epac2 negatively regulates autophagy in an mTOR-independent manner [7, 8]. Therefore, to elucidate the role of mTOR-independent autophagy in brain functioning, including social behaviors, we investigated the functional roles of autophagy pathways in *Epac2*^*−/−*^ mice with social recognition deficiencies. Although the loss of microglial autophagy can be associated with social–behavioral impairments [17], in this study, we focused on neuronal autophagy because no alterations in morphology and number of microglia in the cortex of *Epac2*^*−/−*^ mice were observed (Additional file 1: Fig. S1).

Epac2 deficiencies affect autophagic activity because autophagy is negatively regulated by mTOR and cAMP [18]. Although we examined the cAMP levels, which play an important role in regulating neural autophagic activity and directly activates Epac2 [19], we could not find a significant difference in cAMP levels in the cultured cortical neurons (Additional file 1: Fig. S2). Moreover, when we examined the Rap1 protein expression and enzymatic activity as a downstream signaling pathway of Epac2 activation, we found that the protein expression (Additional file 1: Fig. S3) and enzymatic activity (Additional file 1: Fig. S4) of Rap1 were unchanged in the cortical tissues of Epac2^−/−^ mice in vivo compared with Epac2^+*/*+^ mice. However, calcineurin is activated by lysosomal calcium signaling, which is an endogenous serine/threonine phosphatase that dephosphorylates TFEB, leading to an upregulation of autophagy [6]. We found that a reduction in phosphorylated levels of TFEB in Epac^2−/−^ mice cortex (Additional file 1: Fig. S5). Therefore, altogether, these data suggest that other signaling molecules that are affected by Epac2, such as Ca^2+^, may be involved in the autophagic activity changes of cortical neurons in *Epac2*^*−/−*^ mice via indirect pathways, which are related to neither cAMP nor Rap signaling.

To the best of our knowledge, this is the first report that demonstrates the excessive activity of mTOR-independent autophagy, and Epac2 deficiencies could contribute to defects in social behaviors in mice models. In addition, our study provides therapeutic insights into neurodevelopmental disorder treatment, including ASD with excessive autophagic activity, through suppressing autophagic activity.

## Supplementary Information

**Additional file 1.** Supplemental materials and methods.

**Additional file 2.** Supplemental excel files.

**Additional file 3.** Supplemnetal raw data.

## Data Availability

All data generated or analyzed during this study are included in this published article.

## Abbreviations

ASD: Autism spectrum disorders
ATG: Autophagy-related gene
cAMP: 3′–5′-Cyclic adenosine monophosphate
Epac2: Exchange protein activated by cAMP 2
GABARAPL1: Gamma-aminobutyric acid receptor-associated like protein 1
LC3: Microtubule-associated proteins 1A/1B light chain 3
mTOR: Mammalian target of rapamycin
