# Supplementary material for: Autophagy activity contributes to the impairment of social recognition in Epac2−/− mice
Source: Mol Brain. 2021 Jun 28;14:100. doi: 10.1186/s13041-021-00814-6 (PMC8240198; doi:10.1186/s13041-021-00814-6)
Supplement: Supplementary file 1 — Additional file 1. Supplemental materials and methods. [file 13041_2021_814_MOESM1_ESM.docx]

**Supplementary Information**

**Materials & Methods**

***Animals***

The Epac2 floxed (with PGK-neo) allele was generated by inserting a *lox*P into the 0.5 kb upstream of exon 3 and a FRT-pgk-neo-FRT-loxP cassette into the 0.5 kb downstream of exon 3. This line was generated using MS12 ES cell lines derived from the C57BL/6 strain and maintained in a C57BL/6J genetic background (RIKEN BioResource Research Center, RBRC10748). The Epac2 knockout allele was generated by inducing pCAG-Cre-mediated recombination in the germline of Epac2 floxed mice (RIKEN BioResource Research Center, RBRC10741). Selective deletion of Epac2 mRNA and protein in the brain was verified using qRT-PCR, Western blot, and immunohistochemistry [1]. Here, *Epac2* gene knockout (*Epac2^−/−^*) indicates Epac2A deficiency. Adult male and female mice, between 20 and 50 weeks old, were used for molecular and behavioral experiments. The wild-type (*Epac2^+/+^*) male littermates were used as the control group. Double transgenic *Epac2^−/−^; Atg5^+/−^* mice were used for behavioral tests and immunohistochemistry and were generated by crossing male *Epac^+/−^; Atg5^+/−^* with female *Epac2^+/−^; Atg5^+/−^*. All animals were maintained in a 12-h light–dark cycle and were allowed *ad libitum* access to water and food. The experimental protocol was approved by the Institutional Animal Care and Use Committee at the Kyungpook National University.

***Three-chambered social interaction test***

The three-chambered social apparatus was divided into three equal-size compartments (each chamber-sized: 20 × 40 × 22 cm), by two partitions. In each partition, from the floor to the top, there was a vertical gap (7  × 22 cm) located in the center, allowing access into each chamber. The vertical gaps were initially closed by doors that can be manually opened from the outside. Two small wire cages were placed in each side corner of the apparatus to enclose stranger mice. Mesh wired, sector-shaped containers (radius of 8 cm, angle 90^°^) were attached at the corner of each chamber.

In phase 1, mice were habituated in the center chamber of the apparatus for 10 min. In phase 2, side-chamber doors were opened and the mice were habituated to the whole three-chambered apparatus, containing the two empty cages, for 10 min. In phase 3 for the sociability test, a stranger mouse was placed in one of the two cages while the other cage was left empty. Then, the subject mouse was placed in the center chamber and allowed to freely explore the chamber for 10 min. In phase 4 for the social novelty preference test, a second stranger was placed in the empty cage and the subject mouse could once again freely explore the chamber for 10 min. At the end of each phase, mice were gently guided to move to the center chamber if they were in either the left or right side chambers, and a stranger mouse was introduced to a container, taking approximately 2–3 min. Thus, the subject would have to choose between the mouse previously encountered and a stranger mouse. The time it took for the subject to investigate the first or second stranger mice near the cage was measured. The location of the first stranger mouse was alternated between tests. All the stranger mice used in the test were age- and gender-matched C57BL/6 J mice, which were never previously exposed to the subjects. The apparatus, including the chamber floors, walls, and containers, were cleaned with water and 70% alcohol after each test run. *Epac2^+/+^* (n = 9), *Epac2^−/−^* (n = 10), and *Epac^−/−^; Atg5^+/−^* (n = 8) mice were used for behavioral test.

***Video data analysis of three-chambered social interaction test***

Mouse movement trajectories were obtained from the recorded video files using a customized Matlab program. Briefly, video frames were cropped, grayscaled, and thresholded to acquire binary images. From the binary images, the coordinates of the center of the mouse were extracted and stored serially. Near-container stay duration was computed by constructing 2D image masks (sector radius 10 cm, angle 90^°^) that covered either the left or right container. Frames that contained the coordinates of the center of the mouse within the mask were marked for each phase. Finally, the total near-container stay time was computed by using the ratio of the number of marked frames by the total number of frames, which was then converted into seconds. This was used for subsequent statistical analyses.

***Olfactory habituation/dishabituation test***

To rule out a possibility of impairments in social novelty preference induced by disabilities of social olfactory cues in *Epac2^−/−^* mice, olfactory habituation/dishabituation tests were performed in *Epac2^+/+^*(n = 6), *Epac2^−/−^*(n = 7), and *Epac^−/−^; Atg5^+/−^* (n = 4) mice. Each test mouse was acclimatized in a housing cage with clean bedding for 45 min and a clean, dry cotton tip was used as an odor applicator and was placed in a hole in the lid of the cage during cage habituation. Next, odors (water, almond extract, strawberry extract, unfamiliar social odor 1, and unfamiliar social odor 2) were applied on a cotton tip placed in a hole of the cage lid. Each odor stimulus was presented in three successive trials for 6 min (i.e., 2 min in each trial) with 1 min inter-trial intervals. Almond and strawberry extracts were diluted 1:100 in water and unfamiliar social odors were taken by swabbing the bottom of the unfamiliar mouse’s cage. The cotton tip was placed 2 cm from the mouse, and the sniffing time of each odor was manually measured for each trial for each odor.

***Immunohistochemistry***

Brain sample preparation and immunohistochemistry were performed as previously described [1]. After tissue preparation from *Epac2^+/+^* (n = 3), *Epac2^−/−^* (n = 3), and *Epac^−/−^; Atg5^+/−^* (n = 4) mice (coronal slices, 30 - 40 μm thickness), the slices were permeabilized with 0.1% Triton X-100 at room temperature (RT) for 15 min and were blocked with blocking buffer containing 5% normal goat serum and 0.05% Triton X-100 in PBS at RT for 2 h. The samples were incubated with primary SQSTM1/p62 antibody (1:1,000; H00008878-M01, Abnova) overnight at 4°C or goat anti-ionized calcium-binding adapter protein-1 (Iba1) (1:1000; NB100-1028; Novus Biologicals, Littleton, CO, USA) for 2 days at 4°C. Then, Alexa488-donkey anti-mouse (1:500; 715-545-150, Jackson Immunoresearch) for p62 and Alexa Fluor 488 donkey anti-goat IgG (1:200, Jackson Immunoresearch) for Iba1 were incubated for 2–3 h, at RT. For observation, slices were mounted with DAPI by mounting medium (H-1200, Vectashield). For the detection of p62 signals, images were detected by confocal microscopy (ZEISS, LSM880). For Iba1-positive microglia analysis, images of the immunostained sections (*Epac2^+/+^*(n=5) and *Epac2^−/−^*(n=5)) were acquired with a Zeiss (Thornwood, NY, USA) confocal microscope and an LSM5 EXCITER, version 4.2. Fluorescence images were analyzed using ImageJ (NIH, Bethesda, MD, USA) and MATLAB (Mathworks, Natick, MA, USA).

***Mouse cortical neuron culture and transection***

Cortical neurons from embryonic day 17 (E17) or E18 mice were cultured, as previously described [2]. Briefly, cortical neurons were plated and precoated with poly-L-lysin coverslip in cell culture plates containing Minimum Essential Medium, supplemented with 10% FBS, 0.45% glucose, 2mM L-glutamine, and 1% penicillin/streptomycin, and then maintained in Neurobasal medium supplemented with 2% B-27, 2 mM L-glutamine and 1% penicillin/streptomycin at 37°C in 5% CO_2_. Each plasmid DNA (500 ng to 1 μg) was transfected into primary cortical neuronal cells using Lipofectamine 2000 (Invitrogen, USA), for 1 day *in vitro* (DIV1), according to the manufacturer’s protocol.

***siRNA transfection and Western blot analysis***

For high transfection efficiency during primary neuron culture, siRNAs were inoculated with cells in 6-well plates. The cells were plated at 2 × 10^6^ into a 6-well plate per well and were transfected with siCTL (sc-37007, Santa-Cruz), siATG5 (sc-41446, Santa-Cruz), and siATG7 (sc-41448, Santa-Cruz), each at 20 nM, using RNAiMAX (Lipofectamine™ RNAiMAX Transfection Reagent, 13778150). The medium was changed every 12–16 h. Cells were incubated for 7 days and were treated with CQ (Chloroquine, 50 uM, 24 h, C6628, Sigma). Next, cells were lysed in RIPA buffer with a protease inhibitor cocktail (P8340, Sigma) and phosphatase inhibitor (78441, Thermo) (*Epac2^+/+^*(n = 4), *Epac2^+/+^* +CQ (n = 4), *Epac2^+/+^* + atg5, 7 siRNA(n = 4), *Epac2^+/+^* + atg5, 7 siRNA +CQ (n = 4), *Epac2^−/−^* (n = 4), *Epac2^−/−^* + CQ(n = 4), and *Epac2^−/−^* + atg5, 7 siRNA +CQ (n = 4)). Western blot analysis of mTOR, p70S6 kinase, p-p70S6 kinase using cultured neurons from *Epac2^+/+^*(n = 9) and *Epac2^−/−^* (n = 7) pups was also performed. Western blot analysis of TFEB, pTFEB using cultured neurons from *Epac2^+/+^*(n = 6) and *Epac2^−/−^* (n = 6) pups was also performed. Total 10–20 ug protein was separated on SDS-PAGE and then transferred onto a PVDF membrane (SLHV033RS, Millipore). After blocking with TBST containing 5% non-fat dry skim milk, membranes were incubated with primary antibodies, β-actin (1:10,000, Sigma), LC3B (1:1000, CST, 2775S), GABARAPL1 (1:1000, Genetex, GTX132664), mTOR (1:1000, 215Q18, Thermo), p70S6 kinase (1:1000, #9202, CST), p-p70S6 kinase (1:1000, #9205, CST), ATG5(1:1000, A0856, sigma), TFEB(1:1000, A303-673A, Bethyl laboratory), p-TFEB(S142)(1:1000, ABE1971, Millipore),and ATG7 (1:1000, #2631, CST), overnight at 4°C. Then, the membranes were washed and incubated for 1 h, at RT, with peroxidase-conjugated goat anti-mouse/rabbit antibodies (1:10,000, 115-035-146, and 115-035-174, Jackson Immunoresearch). Signals were detected via chemiluminescence (WBKLS0500, Millipore).

***Autophagy flux assay and quantification of autophagosome numbers***

To examine autophagic flux, cultured neuronal cells at div 1–3 were treated, with or without 50 μM chloroquine (CQ; C6628, Sigma) for 24 h. Cell lysates were used to perform Western blot analyses using anti-LC3, anti-GABARAPL1, and anti-GAPDH antibodies. For the quantification of Western blot for autophagic flux assays, the band intensity of LC3 II, in the presence or absence of CQ in each group, was quantified using Prism software. Data were obtained from three independent experiments (*Epac2^+/+^*(n = 7), *Epac2^+/+^* + CQ(n = 7), *Epac2^−/−^* (n = 7), *Epac2^−/−^* + CQ(n = 7)).

To investigate the autophagosomes in cultured neurons, HyD-LIR-GFP was transfected into cortical neurons at div 1, and 24–48 h after transfection, cells were fixed and HyD-LIR-GFP-positive autophagosomes were counted in cell images taken using confocal laser scanning microscopy (Carl-Zeiss, LSM700). Data were obtained from three independent experiments (*Epac2^+/+^*(n = 9), *Epac2^+/+^* + CQ(n = 10), *Epac2^−/−^*(n = 10), *Epac2^−/−^* + CQ (n = 14)).

***cAMP concentration measurement***

The cortical neurons cultured from *Epac2^+/+^* (n = 6) and *Epac2^−/−^* (n = 5) pups were lysed with 0.1 M HCl buffer and incubated at RT for 20 min for inactivation of phosphodiesterase and immunoglobulins. After centrifugation at top speed for 10 min and removal of any insoluble materials, the concentration of the sample was measured. Before acetylating, diluted cultured neuronal samples (>1 mg/ml) were neutralized with neutralizing buffer, and cAMP contained in the samples was acetylated with an acetylating reagent mix. Following acetylation, samples were diluted with assay buffer. A total of 100 uL of each diluted sample and standard were moved to the protein G coated plate and incubated for 1 h, at RT. Then, a cAMP-HRP solution and HRP-developer were processed, in order, for 1 h. Every reagent used was supplied with the cAMP assay kit (ab65355, Abcam). The cAMP concentration (sample number; *Epac2^+/+^* (n=4) and *Epac2^−/−^* (n=4)) was measured using a microplate reader (SpectraMax190, Molecular devices). Data were obtained from three independent experiments.

***Rap1 protein expression using Western blot***

A total of 15 ug of protein extracted from cortical tissues of *Epac2^+/+^* (n=16) and *Epac2^−/−^* (n=16) was separated on a 12% SDS-PAGE (80 voltage [V] for 30 min, 120 V for 1 hr) and transferred onto a PVDF membrane (SLHV033RS, Millipore). After blocking with TBST containing 5% non-fat dry skim milk, membranes were incubated with primary antibodies against β-actin (1:15,000, Sigma Aldrich #A5441) and Rap1 (1:1000, Cell Biolabs #240602) overnight at 4°C. Membranes were subsequently washed and incubated for 1 h, at RT, with peroxidase-conjugated donkey anti-mouse/goat antibodies (1:5,000, Bethyl #A90-116P/#A50-101P). Signals were detected by chemiluminescence (ECL blotting reagent, GE Healthcare #RPN2109).

***Pull-down assay for Rap1 enzymatic activity***

The concentration of all samples (*Epac2^+/+^* (n=12) and *Epac2^−/−^* (n=8)) was adjusted to 5 mg/ml. Both GTPγS (240103, Cell Biolabs) and GDP (240104, Cell Biolabs) were loaded to brain lysates of BL6/J naive mice for the positive and negative controls. A volume of 20 uL of 0.5 M EDTA and 10 uL of 100 X GTPγS (positive control)/GDP (negative control) were added into 1 mL experimental samples. The mixtures were incubated with agitation for 30 min at 30°C. The GTPγS/GDP loading process was stopped by adding 65 uL of 1 M MgCl2 to each tube, which was placed on ice. To the samples, 40 uL of 50% agarose bead slurry with Rap-binding domain of murine RalGDS (STA-418, Cell Biolabs) was mixed by vortex in 1 mL of samples, and then treated with either Rap activators or left untreated. The beads-lysate mixtures were incubated at 4°C for 1 h with gentle agitation. Samples were centrifuged for 10 sec at 14000 g, and the supernatants were discarded. The pellet of beads was resuspended in 40 uL of 2 X reducing SDS-PAGE sample buffer and were subsequently boiled for 5 min and loaded onto a Western blot with the same procedures as described in the Western blot for Rap1 protein expression.

***Statistics***

Statistical analyses were performed using Matlab, SPSS25, and Prism5 software. The data of HyD-LIR-GFP-positive autophagosomes and Western blot data for LC3-II and GABARAPL1-II were analyzed by one-way ANOVA and then Tukey’s Multiple Comparison Test. Data for the cAMP measurement; Western blot data for mTOR-dependent pathway and Rap1 protein expression; pull-down assay for Rap1 enzymatic activity, phosphorylated level of TFEB, and microglial cell number were all analyzed using t-test. For a three-chambered social interaction test, a two-sided paired t-test was used to compare the average time spent near the container of either stranger or empty chambers. For an olfactory habituation/dishabituation test, repeated measures ANOVA (mixed model) was used (between-subject factor, genotype; within-subject factor, odor) and Huynh-Feldt corrected degree of freedom was applied if Mauchly’s sphericity test was not satisfied. For quantification and statistical analysis of p62 aggregation in brain tissue, p62 aggregates with >1 μm (diameter) were quantified, and one-way ANOVA and Tukey’s Multiple Comparison Test were used. Data were obtained from three different animals both the WT and *Epac2^−/−^* mice. Data were represented as mean ± the standard error of the mean (SEM), and p-values < 0.05 were considered statistically significant.

**References**

1. Lee K, Kobayashi Y, Seo H, Kwak JH, Masuda A, Lim CS, Lee HR, Kang SJ, Park P, Sim SE, et al: **Involvement of cAMP-guanine nucleotide exchange factor II in hippocampal long-term depression and behavioral flexibility.** *Mol Brain* 2015, **8:**38.

2. Jun MH, Ryu HH, Jun YW, Liu T, Li Y, Lim CS, Lee YS, Kaang BK, Jang DJ, Lee JA: **Sequestration of PRMT1 and Nd1-L mRNA into ALS-linked FUS mutant R521C-positive aggregates contributes to neurite degeneration upon oxidative stress.** *Sci Rep* 2017, **7:**40474.

**Supplementary Figures & Legends**

**
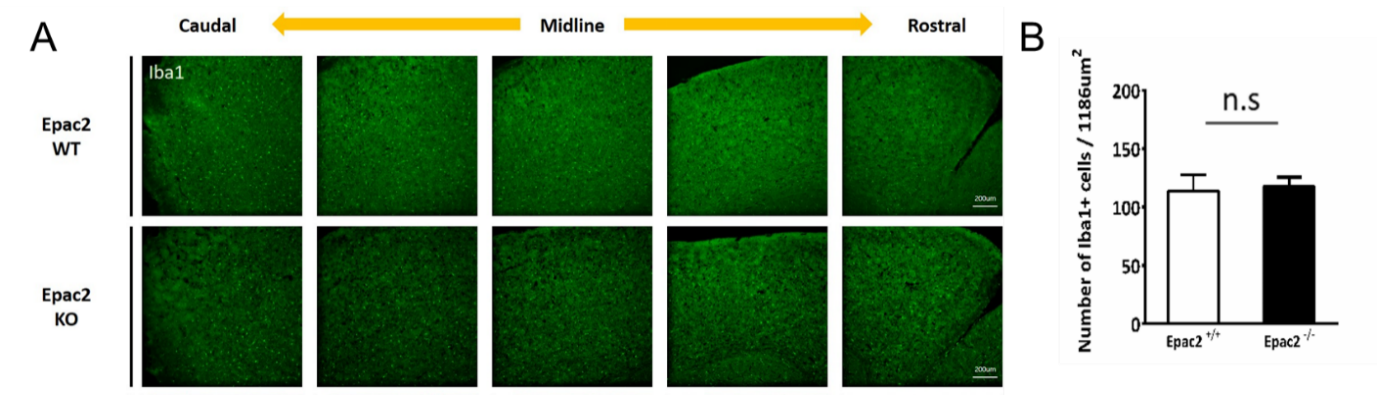
Supplementary Figure 1. Microglia in the cortices of *Epac2^+/+^* and *Epac2^−/−^* mice.** (A) Immunofluorescence images of Iba-1 as a microglial marker in the caudal to rostral cortex. Scale bars, 200 um. (B) Number of Iba-1-immunopositive microglia per area. Note that no difference in the number of microglia between genotypes was observed. *Epac2^+/+^*(n=5) and *Epac2^−/−^*(n=5).

**
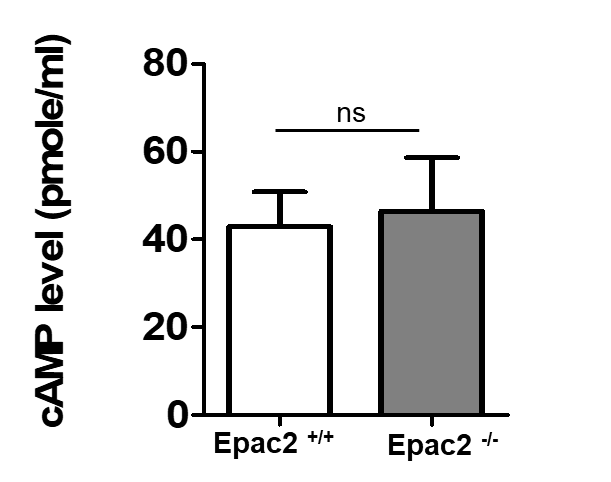
**

**Supplementary Figure 2. cAMP level in cortical neurons cultured from *Epac2^+/+^* and *Epac2^−/−^* pups.** There is no significant difference in the level of cAMP between WT and *Epac2^−/−^* cultured cortical neurons. Sample number; *Epac2^+/+^* (n=4) and *Epac2^−/−^* (n=4), ns, not significant.

**
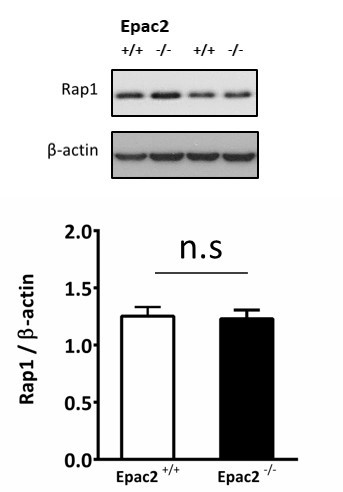
**

**Supplementary Figure 3. Rap1 protein expression in cortical tissues of *Epac2^+/+^* and *Epac2^−/−^* mice.** There is no significant difference in the Rap1 protein expression level between the two genotypes. ns, not significant.

**
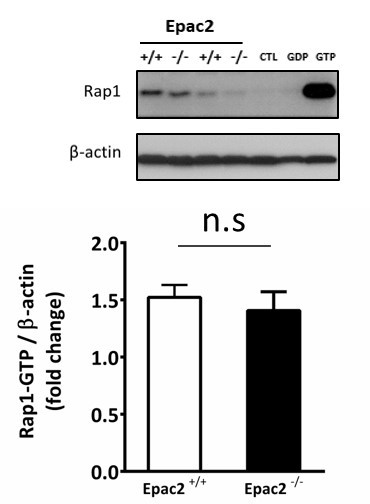
**

**Supplementary Figure 4. Rap1 enzymatic activity in cortical tissues of *Epac2^+/+^* and *Epac2^−/−^* mice.** There is no significant difference in the Rap1 protein activity between the two genotypes. ns, not significant.


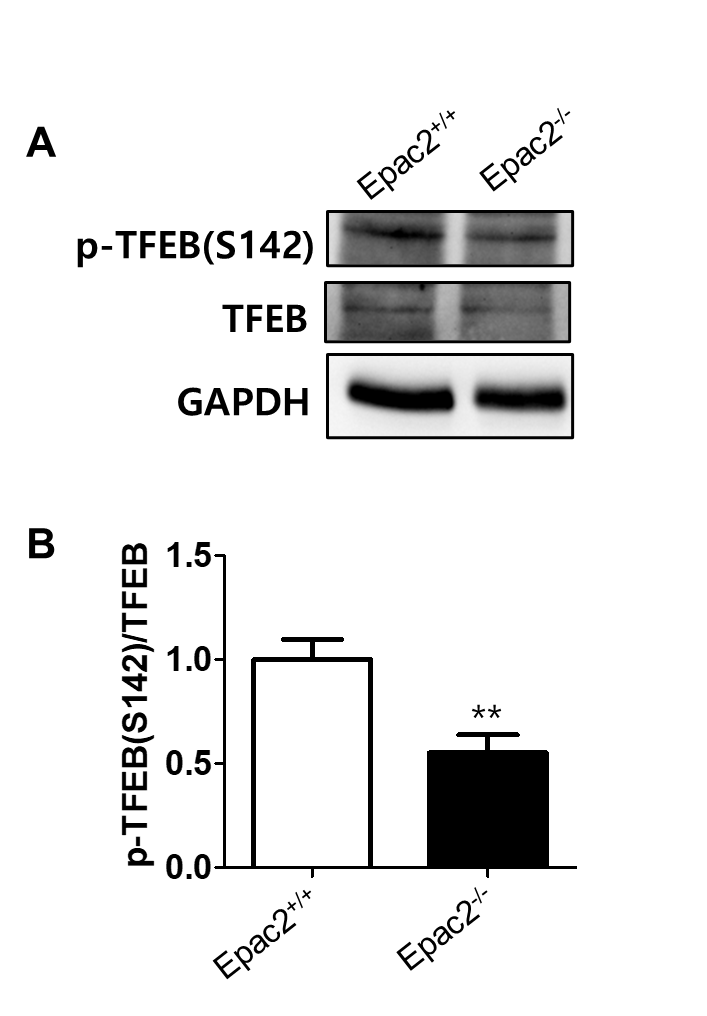


**Supplementary Figure 5. Phosphorylation of TFEB in cortical tissues of *Epac2^+/+^* (n=6) and *Epac2^−/−^* mice (n=6). (A-B)** Phosphorylated levels of TFEB were reduced in *Epac2^−/−^* mice cortex, Student T-test, p=0.0061.
