# Supplementary material for: Autophagy activity contributes to the impairment of social recognition in Epac2−/− mice
Source: Mol Brain. 2021 Jun 28;14:100. doi: 10.1186/s13041-021-00814-6 (PMC8240198; doi:10.1186/s13041-021-00814-6)
Supplement: Supplementary file 3 — Additional file 3. Supplemnetal raw data. [file 13041_2021_814_MOESM3_ESM.pdf]

# Fig1A

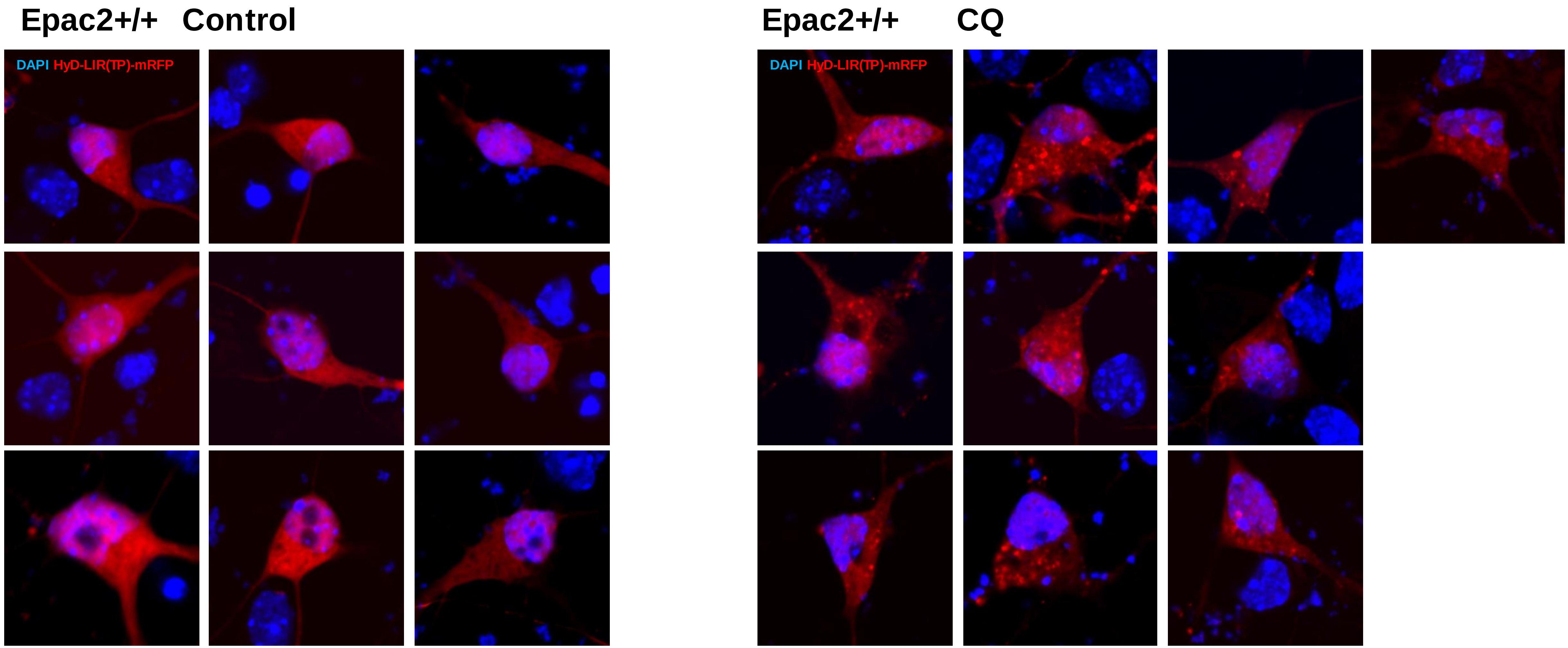
Epac2+/+ Control
DAPI HyD-LIR(TP)-mRFP
Epac2+/+ CQ
DAPI HyD-LIR(TP)-mRFP

Fig1A

Epac2-/- Control

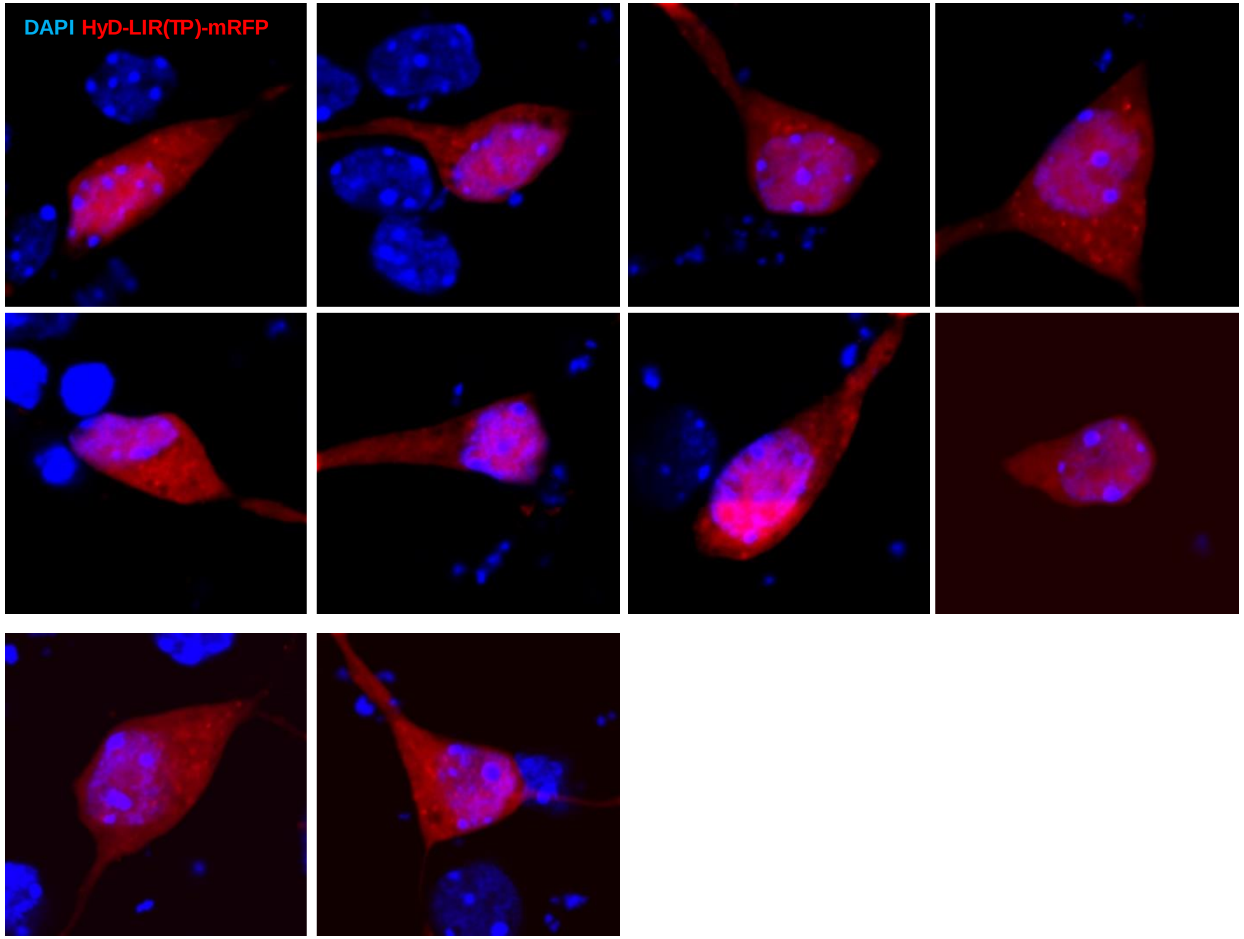


Epac2-/- CQ

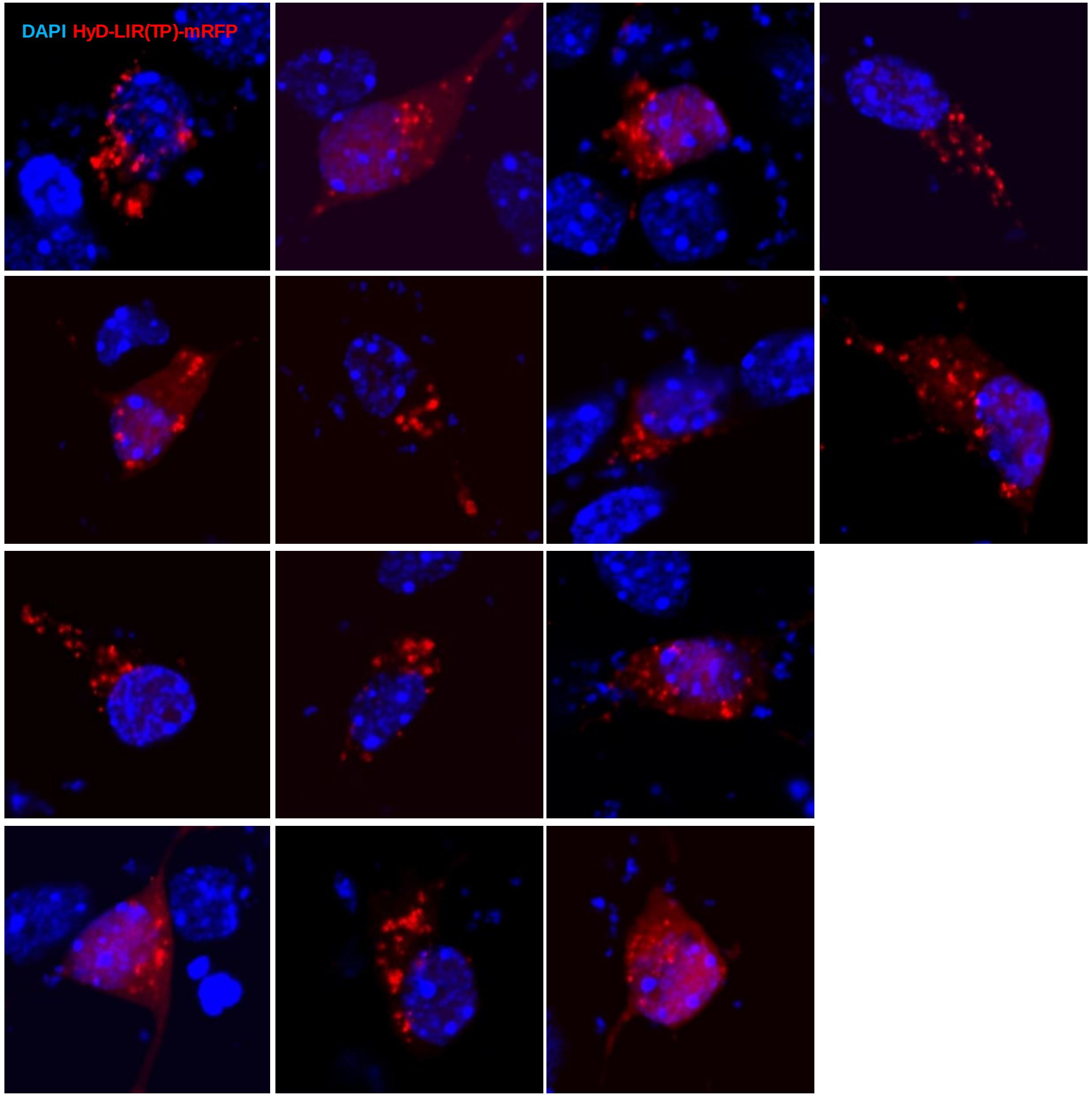

Fig1C

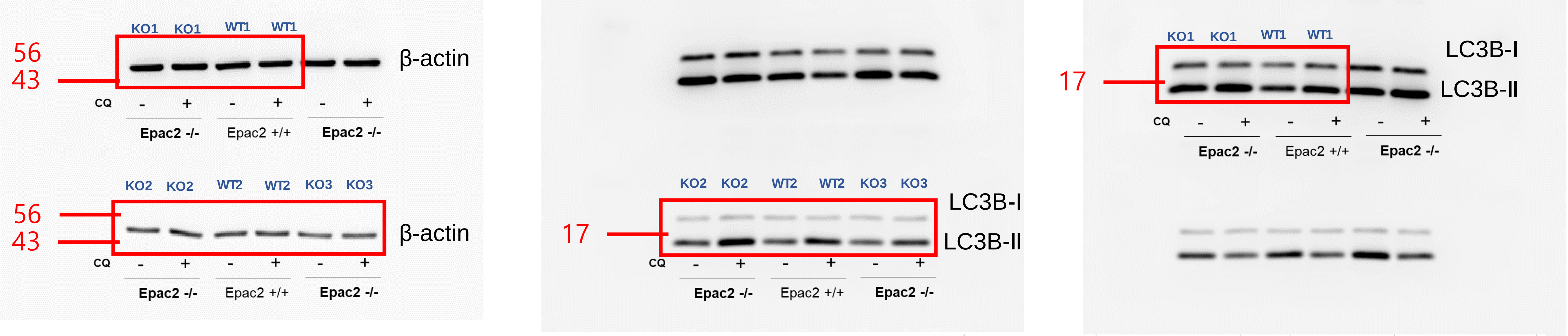


| WT | |
|---|---|
| sample ID | Mouse ID |
| WT1_16wks | 16W WT G40 |
| WT2_16wks | 16W WT G41 |
| WT5_over 17ws | 17W WT G856 |
| WT7_over 17ws | 34W WT G1+ G3 |
| WT8_over 17ws | 54W WT G1106 + G1108 |

| KO | |
|---|---|
| sample ID | Mouse ID |
| KO1_16wks | 16W KO G868 |
| KO2_16wks | 16W KO G869 |
| KO3_16wks | 16W KO G36 |
| KO5_over 17ws | 17W KO G864 |
| KO6_over 17ws | 19W KO G38 + G39 |
| KO7_over 17ws | 29W KO G835 |
| KO8_over 17ws | 54W KO G1044+ G1115 |

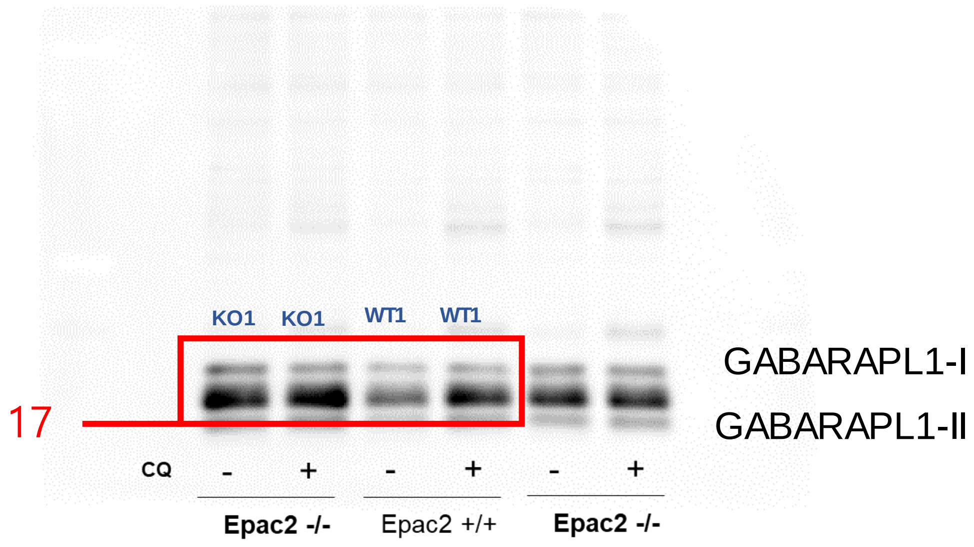


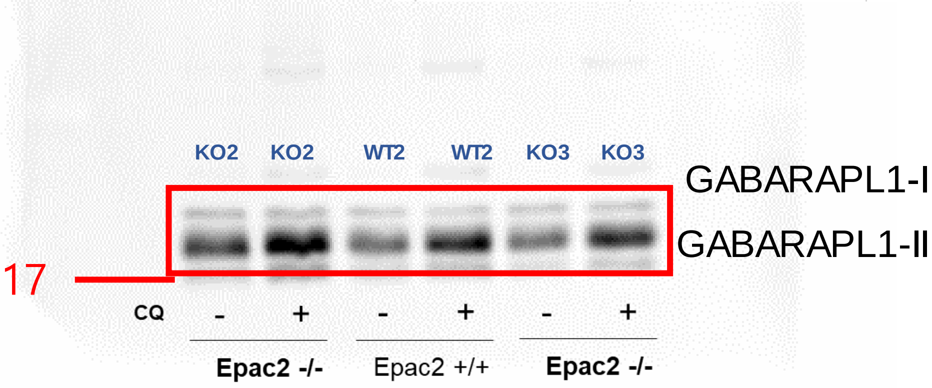

**Fig1C**

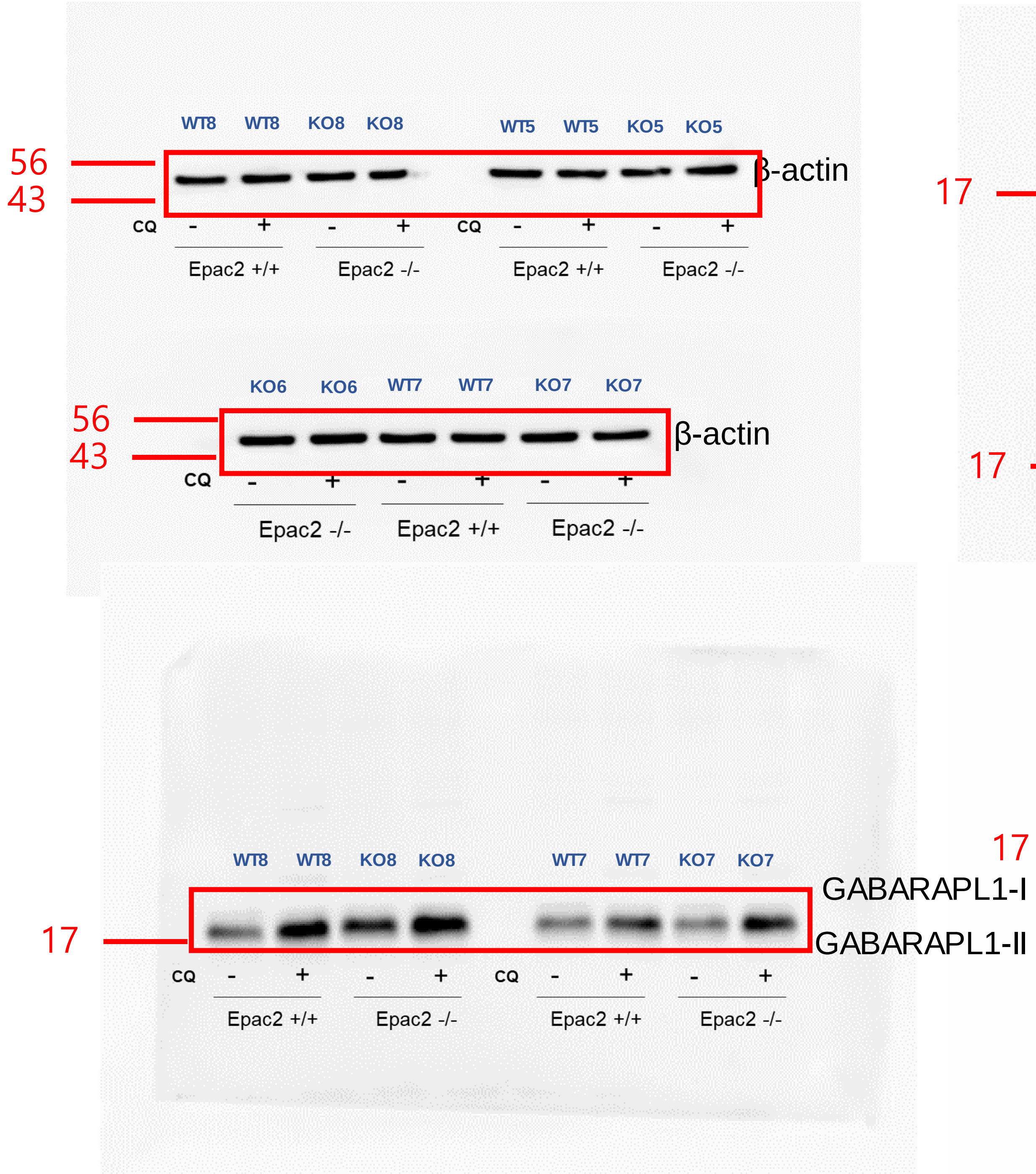


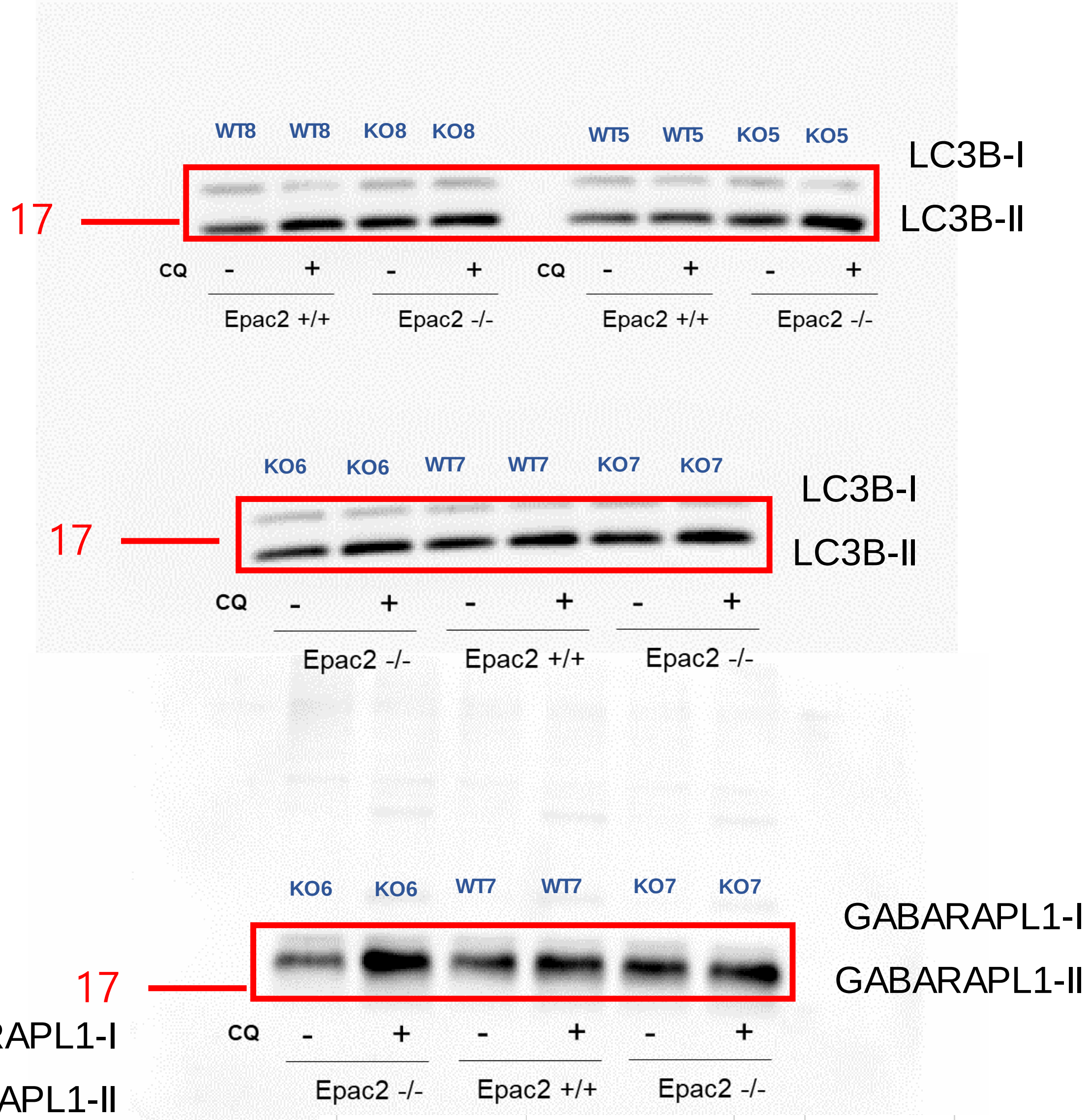


| WT | |
|---|---|
| sample ID | Mouse ID |
| WT1_16wks | 16W WT G40 |
| WT2_16wks | 16W WT G41 |
| WT5_over 17ws | 17W WT G856 |
| WT7_over 17ws | 34W WT G1+ G3 |
| WT8_over 17ws | 54W WT G1106 + G1108 |

| KO | |
|---|---|
| sample ID | Mouse ID |
| KO1_16wks | 16W KO G868 |
| KO2_16wks | 16W KO G869 |
| KO3_16wks | 16W KO G36 |
| KO5_over 17ws | 17W KO G864 |
| KO6_over 17ws | 19W KO G38 + G39 |
| KO7_over 17ws | 29W KO G835 |
| KO8_over 17ws | 54W KO G1044+ G1115 |

# Fig1F

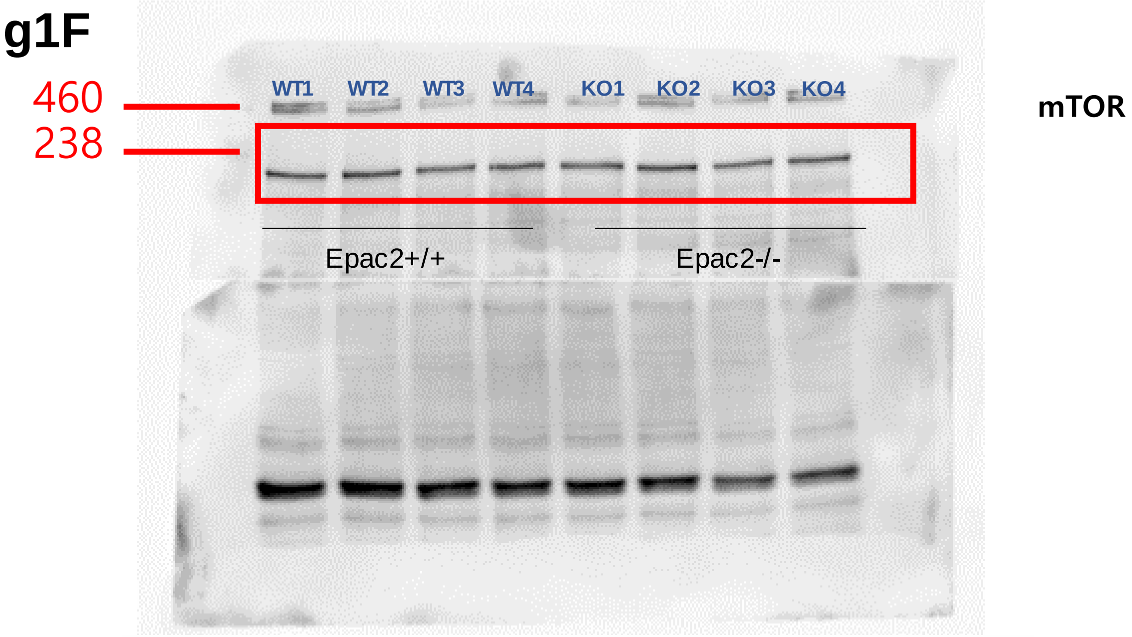


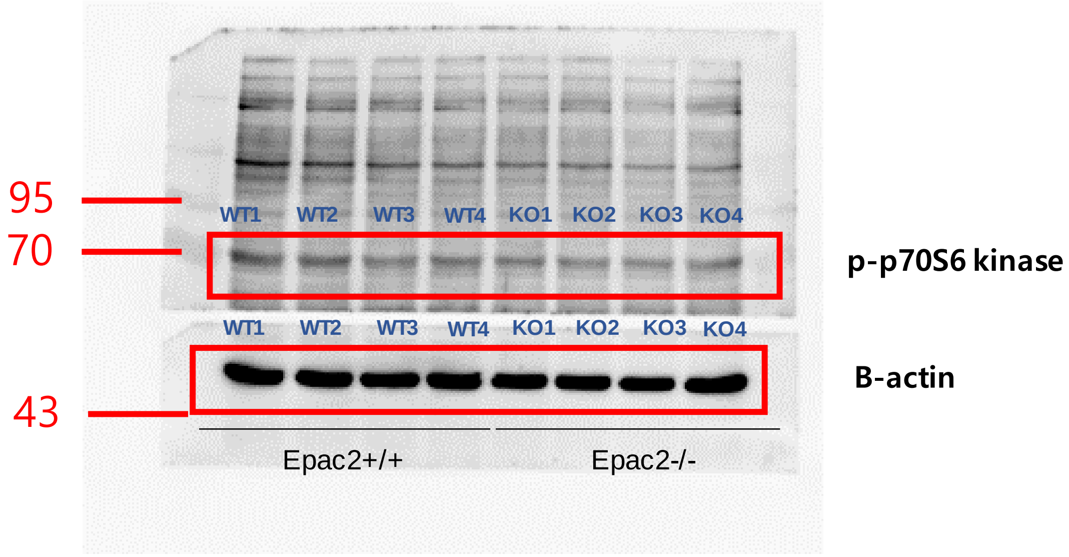


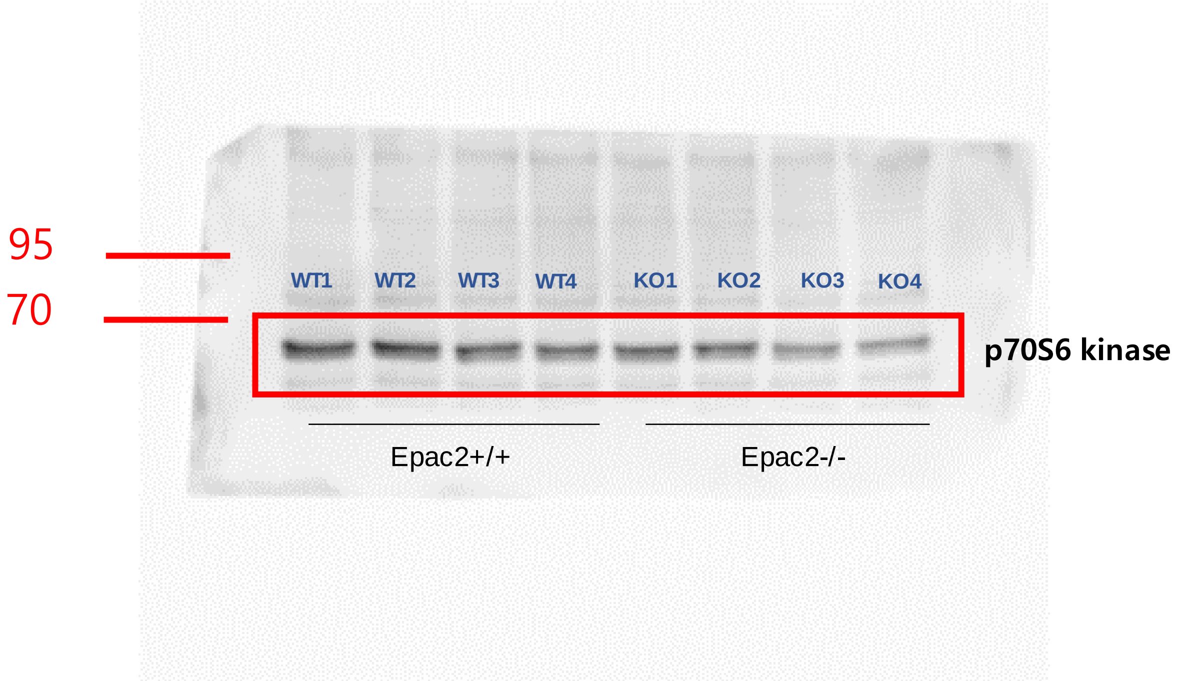


| WT | |
|---|---|
| sample ID | Mouse ID |
| WT1_16wks | 16W WT G40 |
| WT2_16wks | 16W WT G41 |
| WT3_16wks | 16W WT G45 |
| WT4_16wks | 16W WT G57 |
| WT5_over 17ws | 17W WT G856 |
| WT6_over 17ws | 19W WT G854 |
| WT7_over 17ws | 34W WT G1+ G3 |
| WT8_over 17ws | 54W WT G1106 + G1108 |
| WT9_over 17ws | 21W WT G859 |

| KO | |
|---|---|
| sample ID | Mouse ID |
| KO1_16wks | 16W KO G868 |
| KO2_16wks | 16W KO G869 |
| KO3_16wks | 16W KO G36 |
| KO4_16wks | 16W KO G60 |
| KO5_over 17ws | 17W KO G864 |
| KO6_over 17ws | 19W KO G38 + G39 |
| KO7_over 17ws | 29W KO G835 |
| KO8_over 17ws | 54W KO G1044+ G1115 |

# Fig1F

| WT | |
|---|---|
| sample ID | Mouse ID |
| WT1_16wks | 16W WT G40 |
| WT2_16wks | 16W WT G41 |
| WT3_16wks | 16W WT G45 |
| WT4_16wks | 16W WT G57 |
| WT5_over 17ws | 17W WT G856 |
| WT6_over 17ws | 19W WT G854 |
| WT7_over 17ws | 34W WT G1+ G3 |
| WT8_over 17ws | 54W WT G1106 + G1108 |
| WT9_over 17ws | 21W WT G859 |

| KO | |
|---|---|
| sample ID | Mouse ID |
| KO1_16wks | 16W KO G868 |
| KO2_16wks | 16W KO G869 |
| KO3_16wks | 16W KO G36 |
| KO4_16wks | 16W KO G60 |
| KO5_over 17ws | 17W KO G864 |
| KO6_over 17ws | 19W KO G38 + G39 |
| KO7_over 17ws | 29W KO G835 |
| KO8_over 17ws | 54W KO G1044+ G1115 |

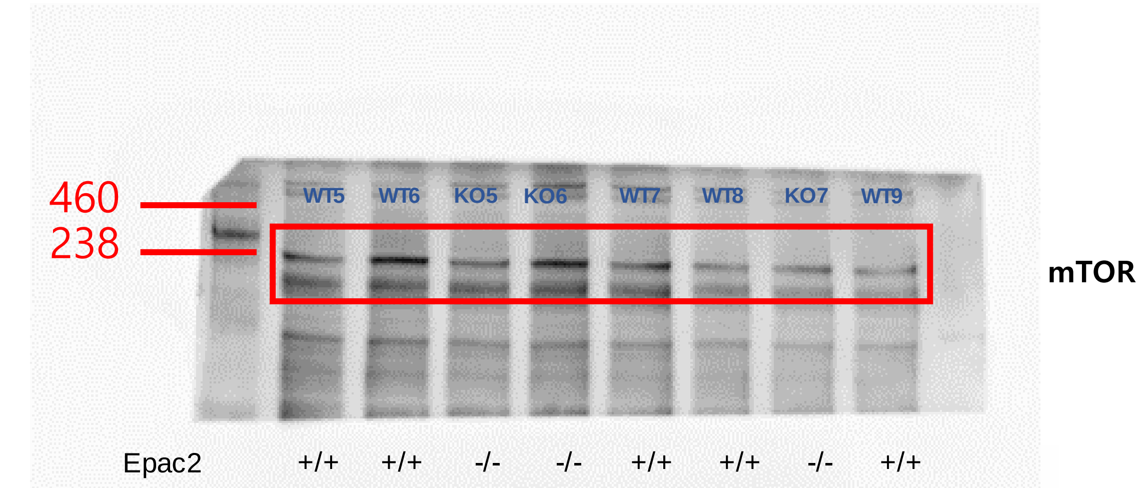


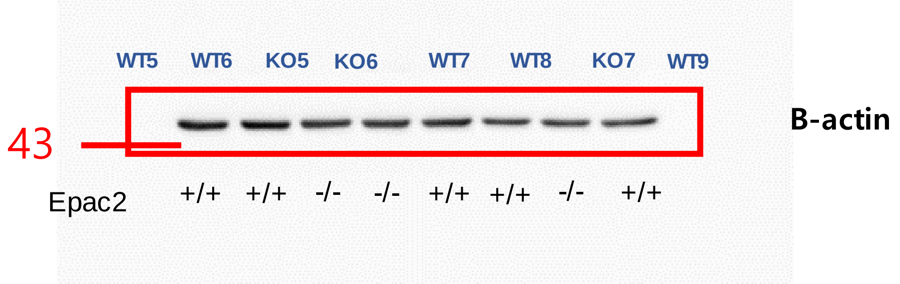


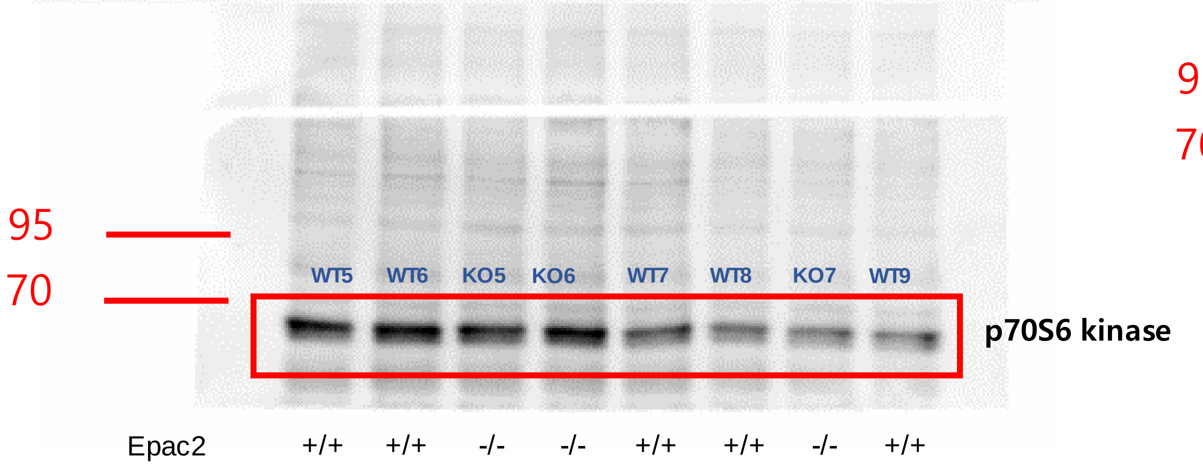


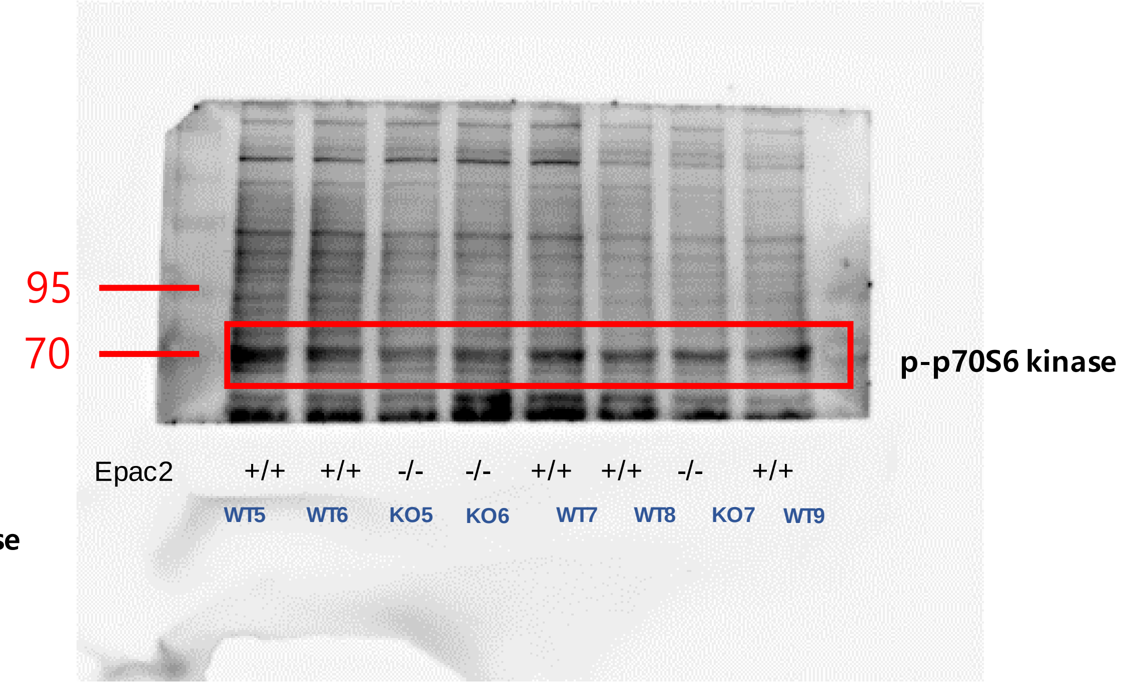

Fig1K

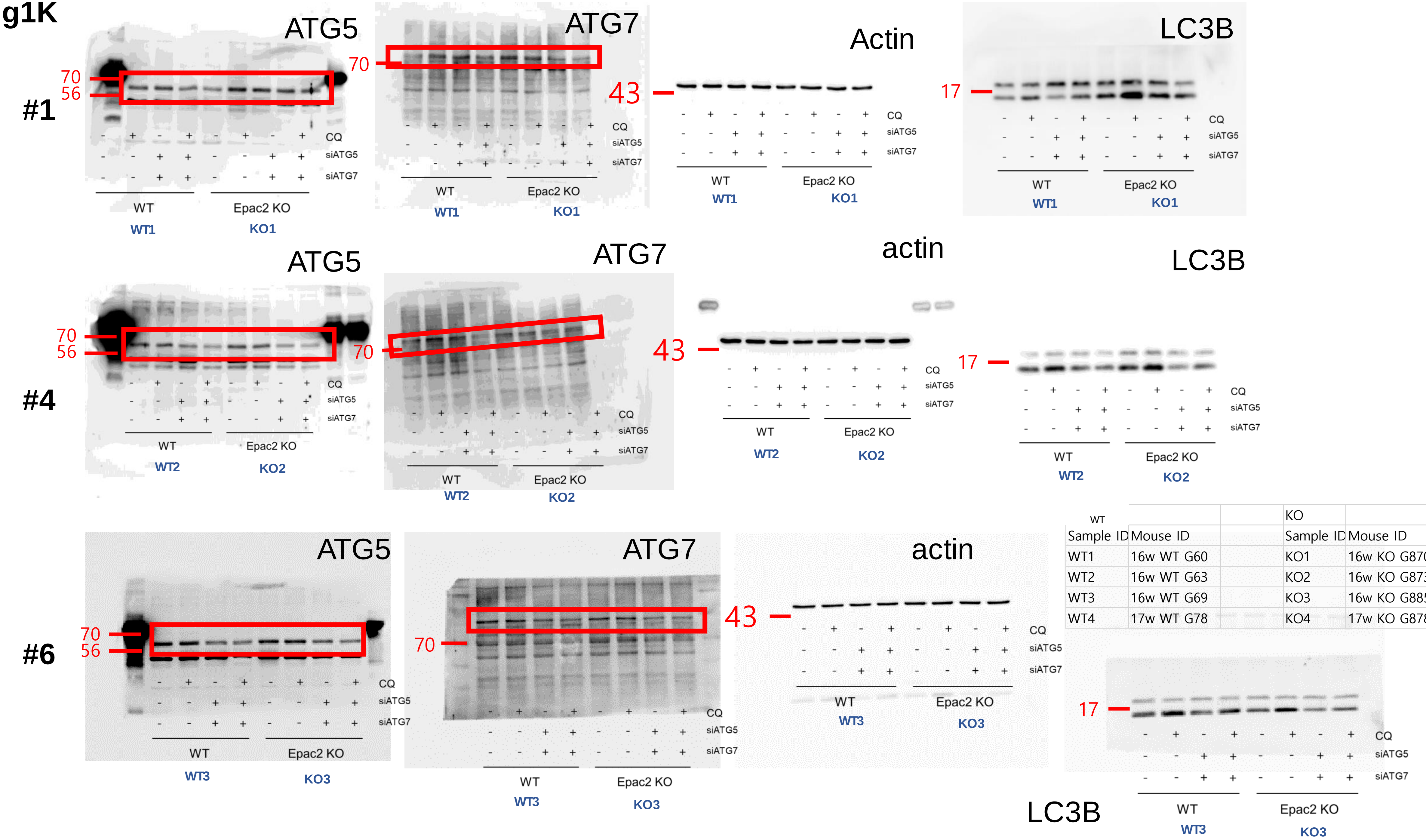


| WT | | | KO | |
|---|---|---|---|---|
| Sample ID | Mouse ID | | Sample ID | Mouse ID |
| WT1 | 16w WT G60 | | KO1 | 16w KO G870 |
| WT2 | 16w WT G63 | | KO2 | 16w KO G873 |
| WT3 | 16w WT G69 | | KO3 | 16w KO G885 |
| WT4 | 17w WT G78 | | KO4 | 17w KO G878 |

**Fig1K**

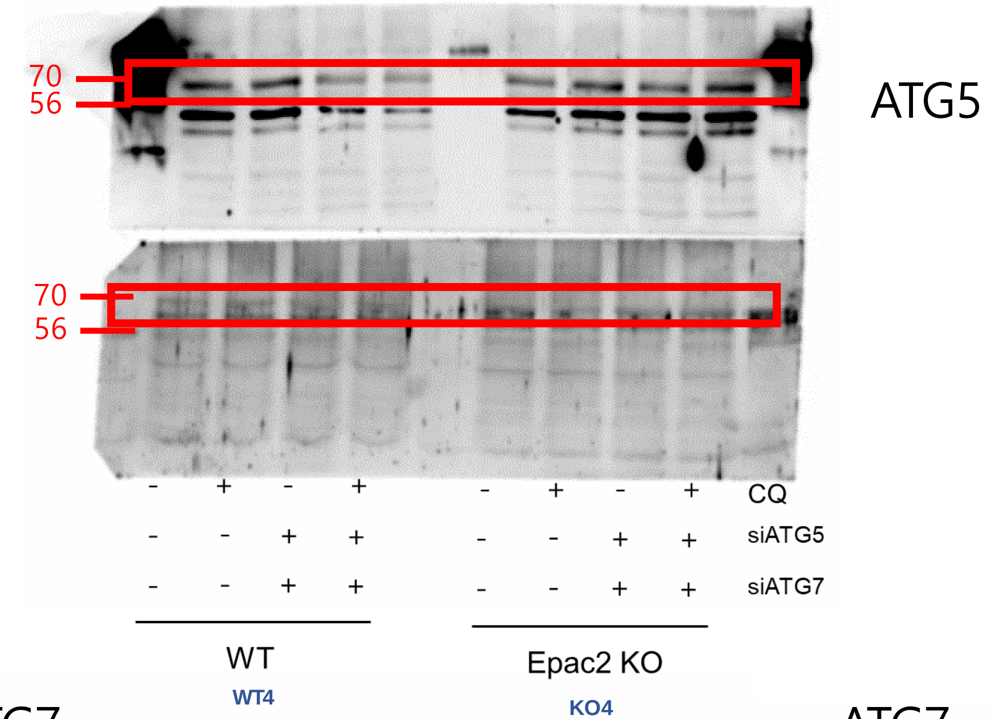


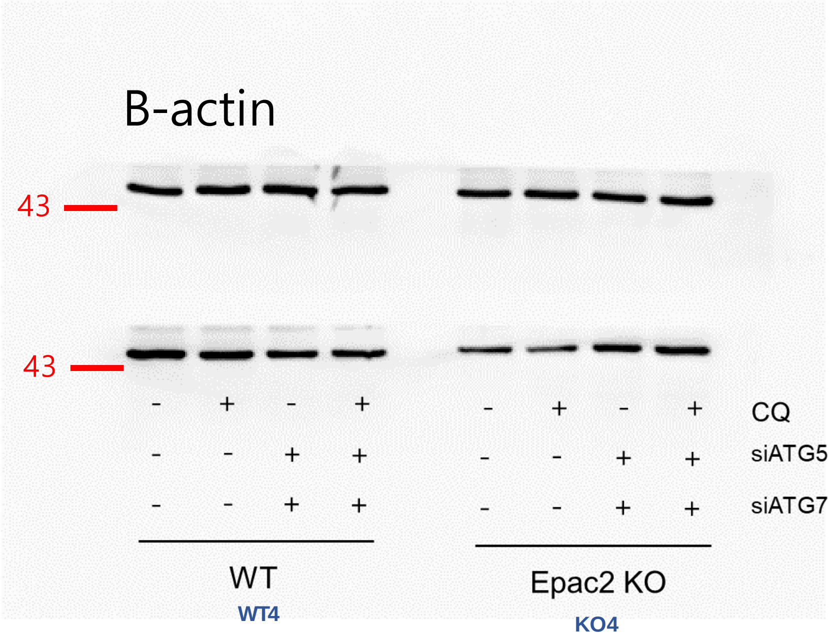


| WT | | | KO | |
|---|---|---|---|---|
| Sample ID | Mouse ID | | Sample ID | Mouse ID |
| WT1 | 16w WT G60 | | KO1 | 16w KO G870 |
| WT2 | 16w WT G63 | | KO2 | 16w KO G873 |
| WT3 | 16w WT G69 | | KO3 | 16w KO G885 |
| WT4 | 17w WT G78 | | KO4 | 17w KO G878 |

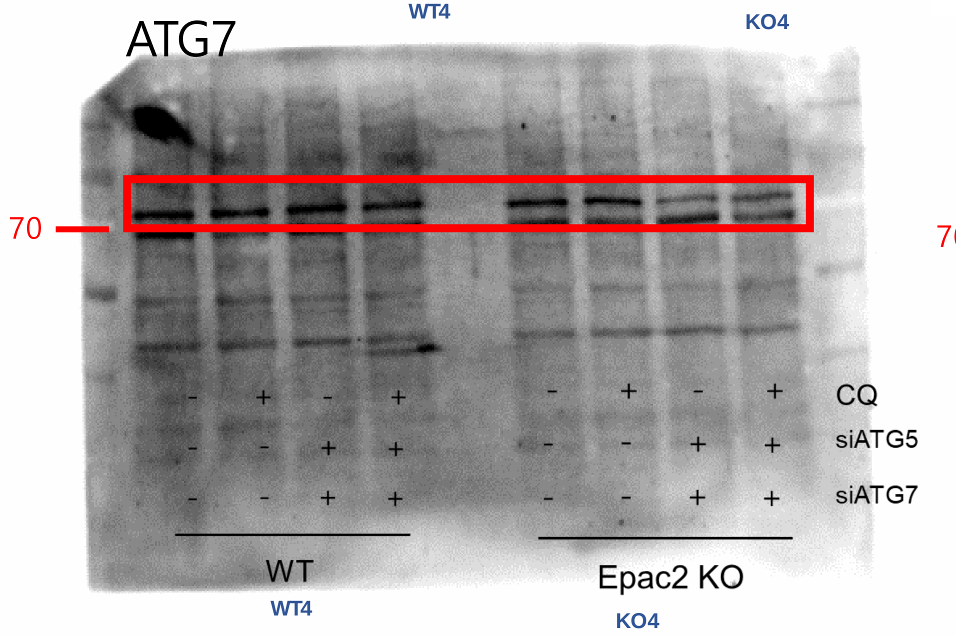


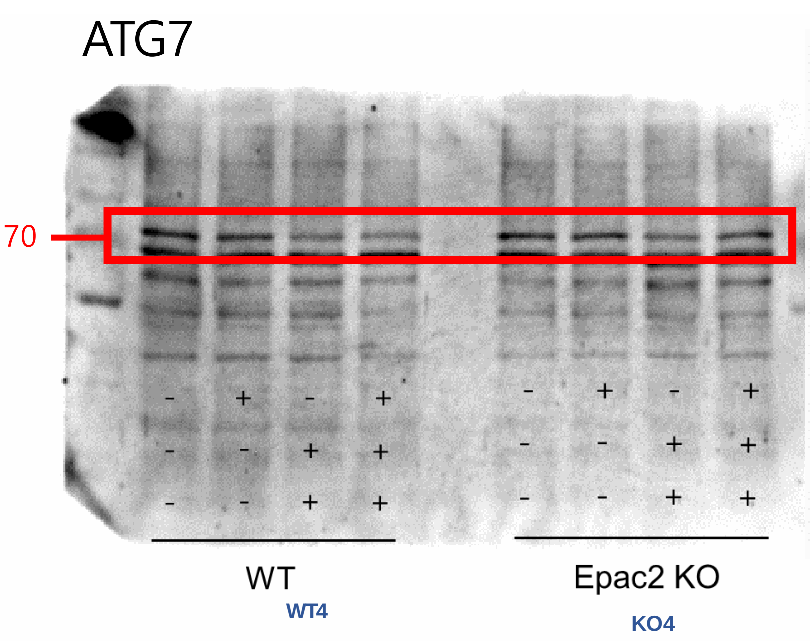


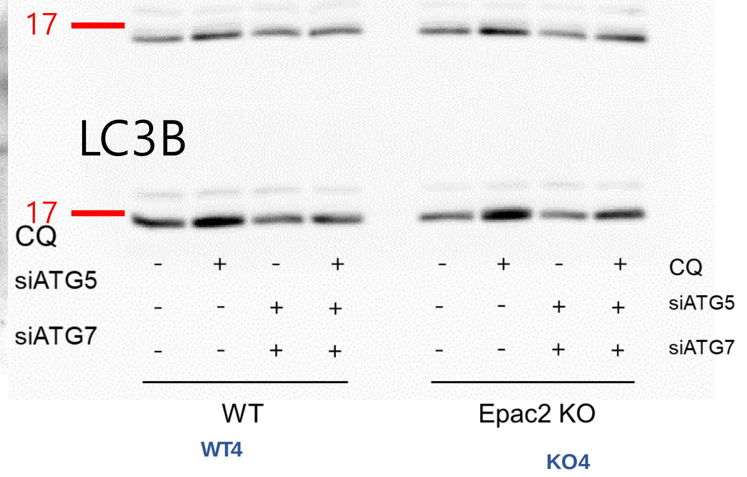

Fig1M

Epac2+/+

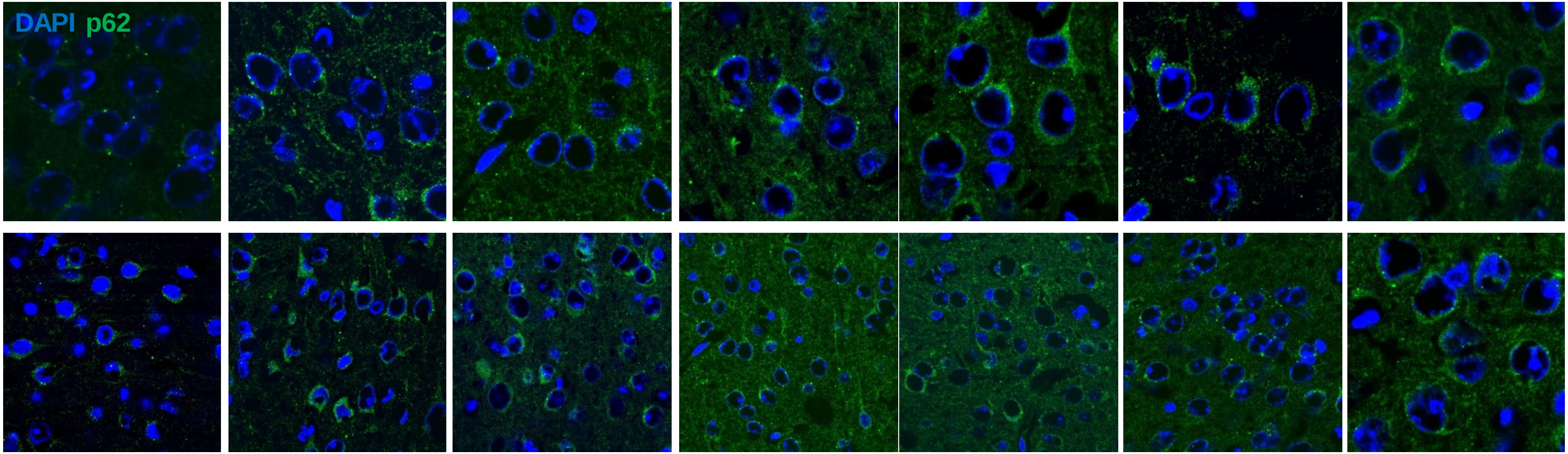

# Fig1M

## Epac2-/-

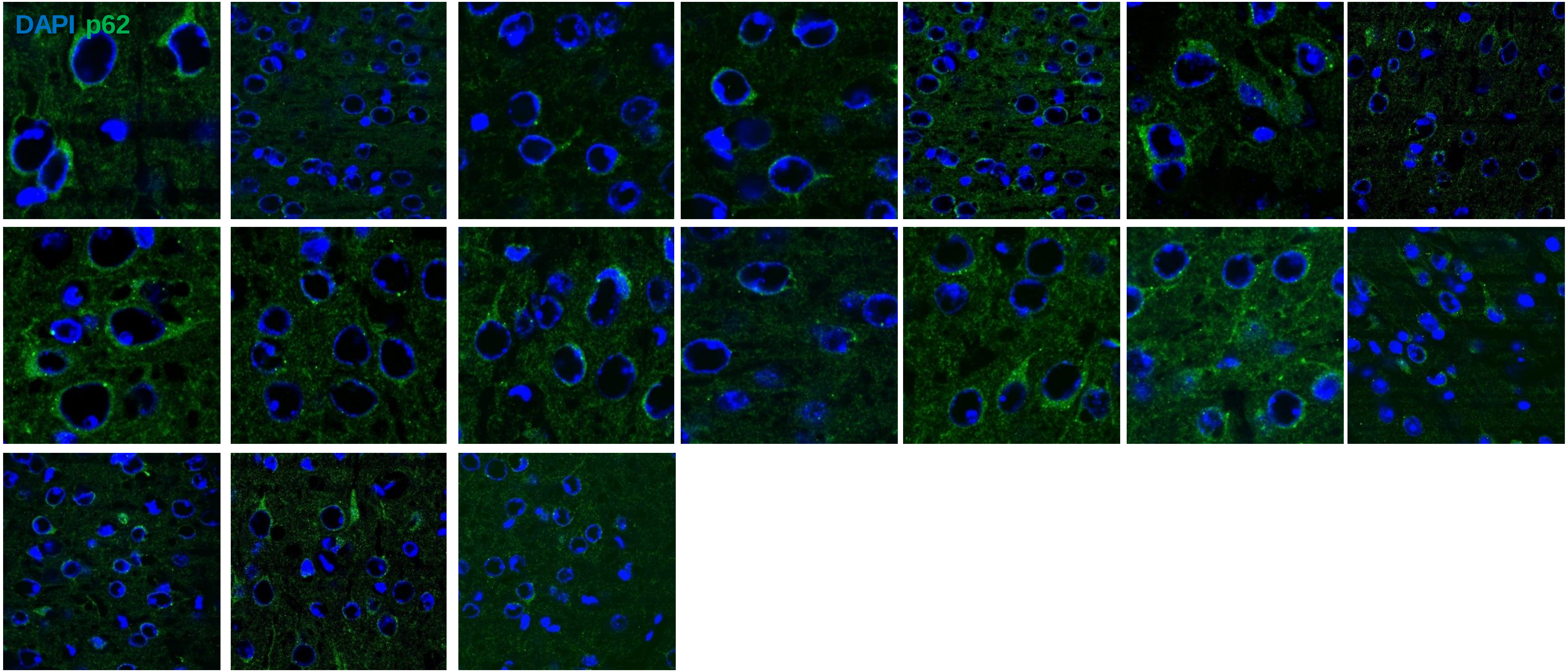

Fig1M

Epac2-/- ; ATG5

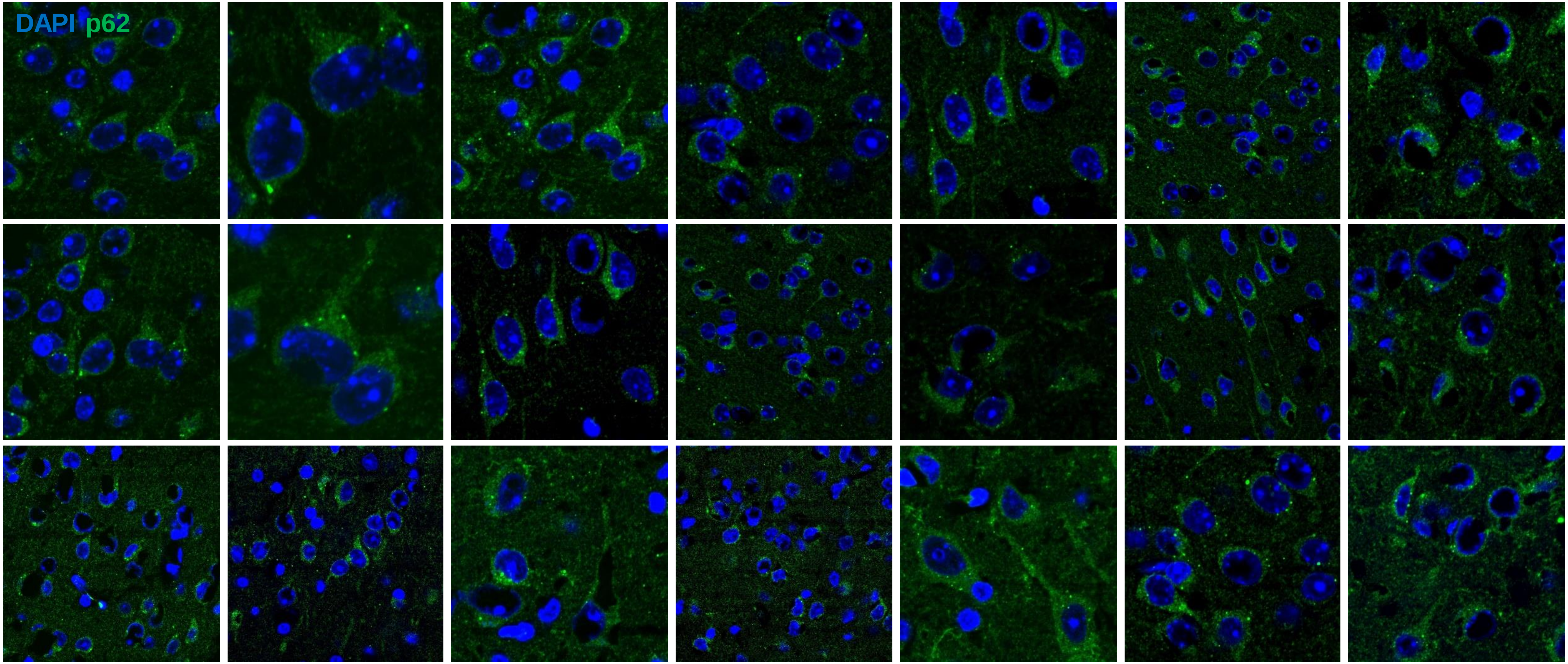

**Supple 1**

Epac2 WT_caudal1 50x

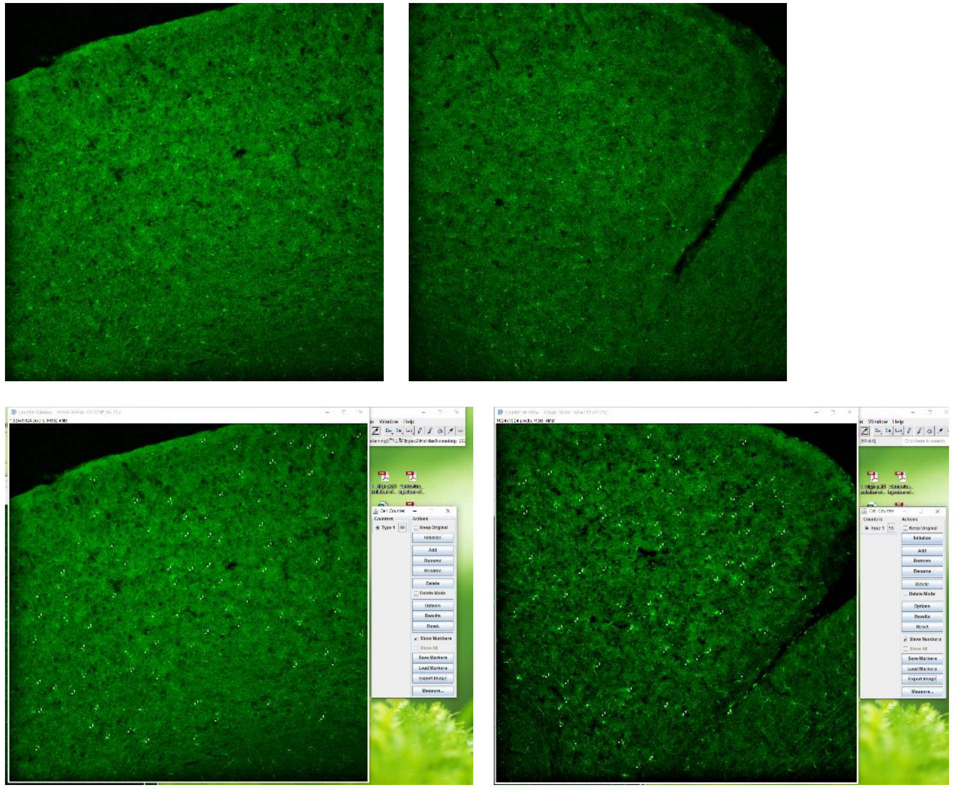

Epac2 WT_mid 50x

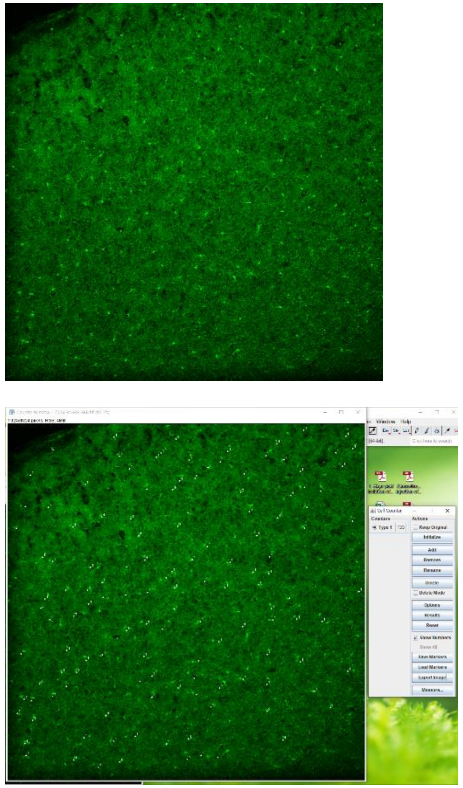

Epac2 WT_rostral1 50x

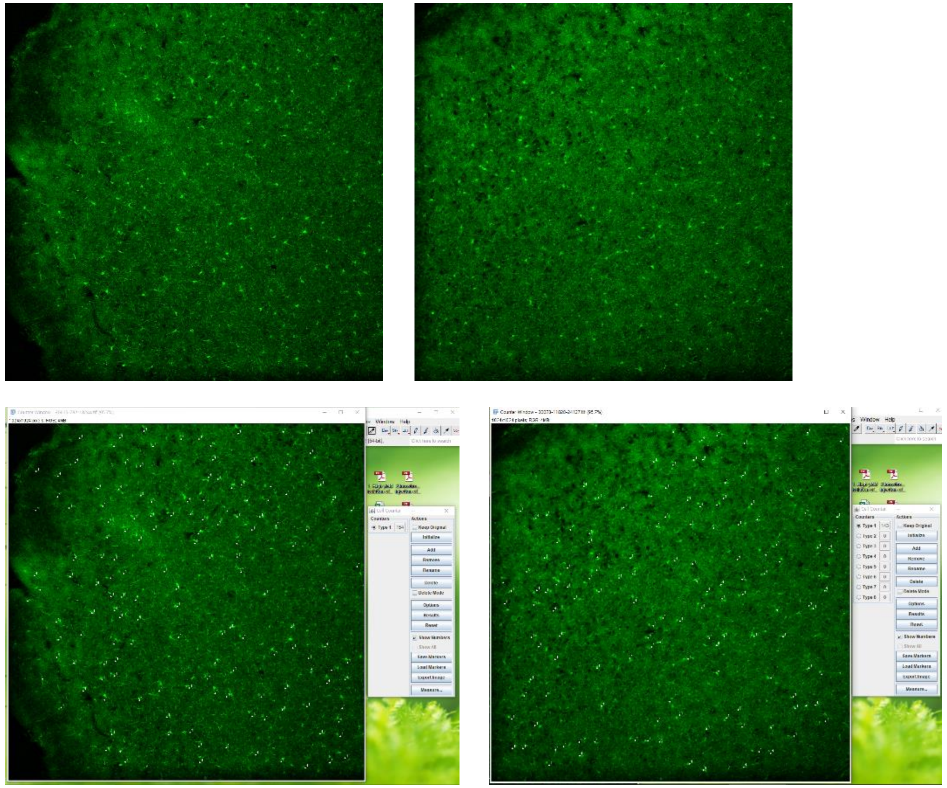

# Supple 1

Epac2 KO_caudal1 50x

Epac2 KO_MID 50x

Epac2 KO_rostral1 50x

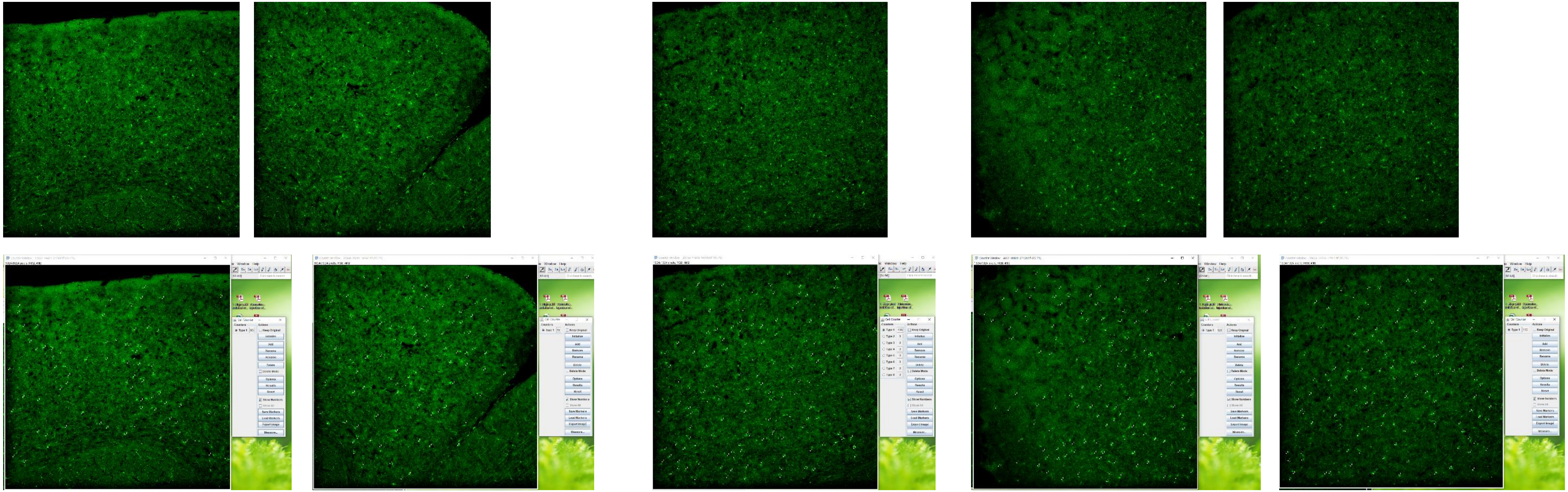

# Supple 3

Trial 1

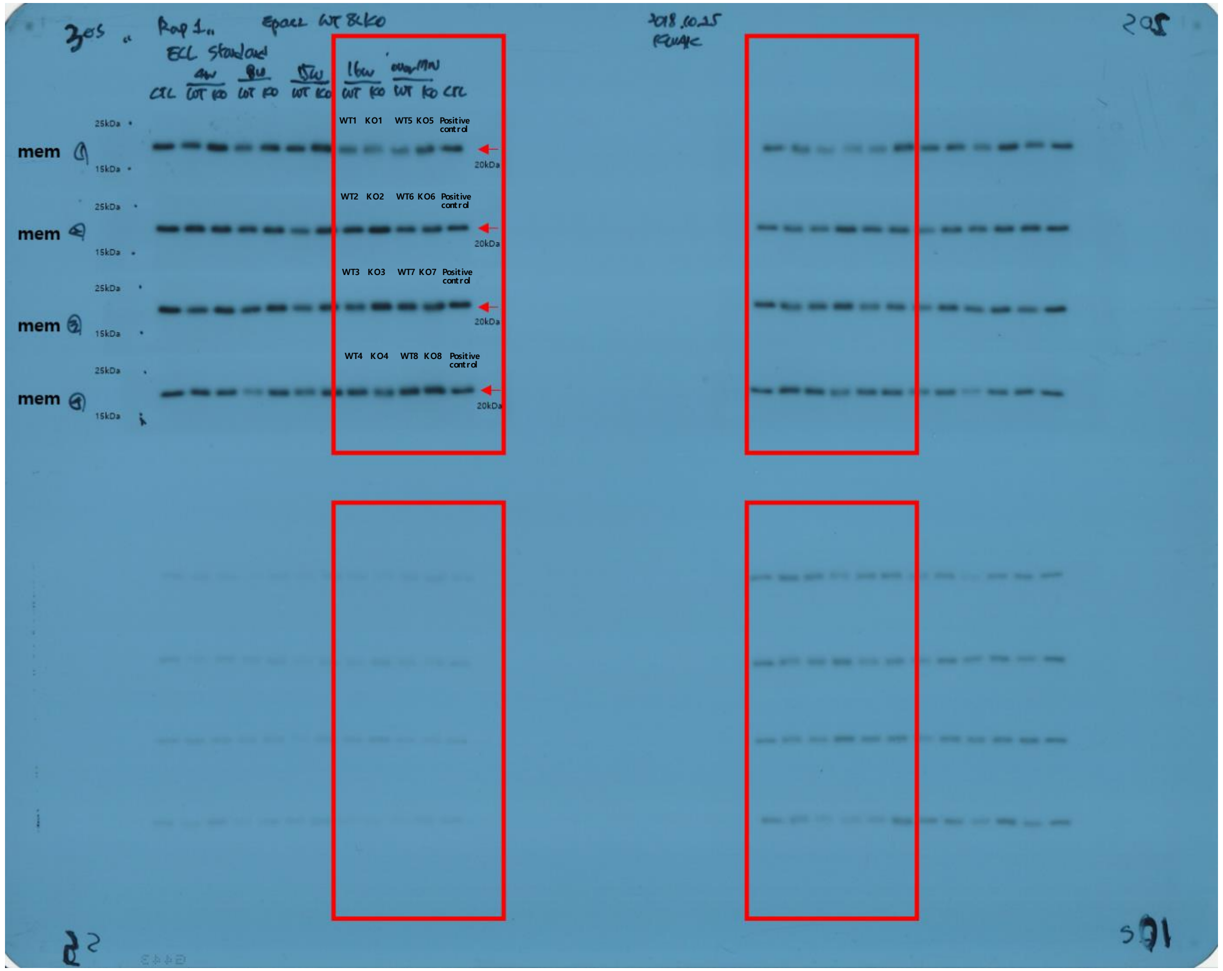


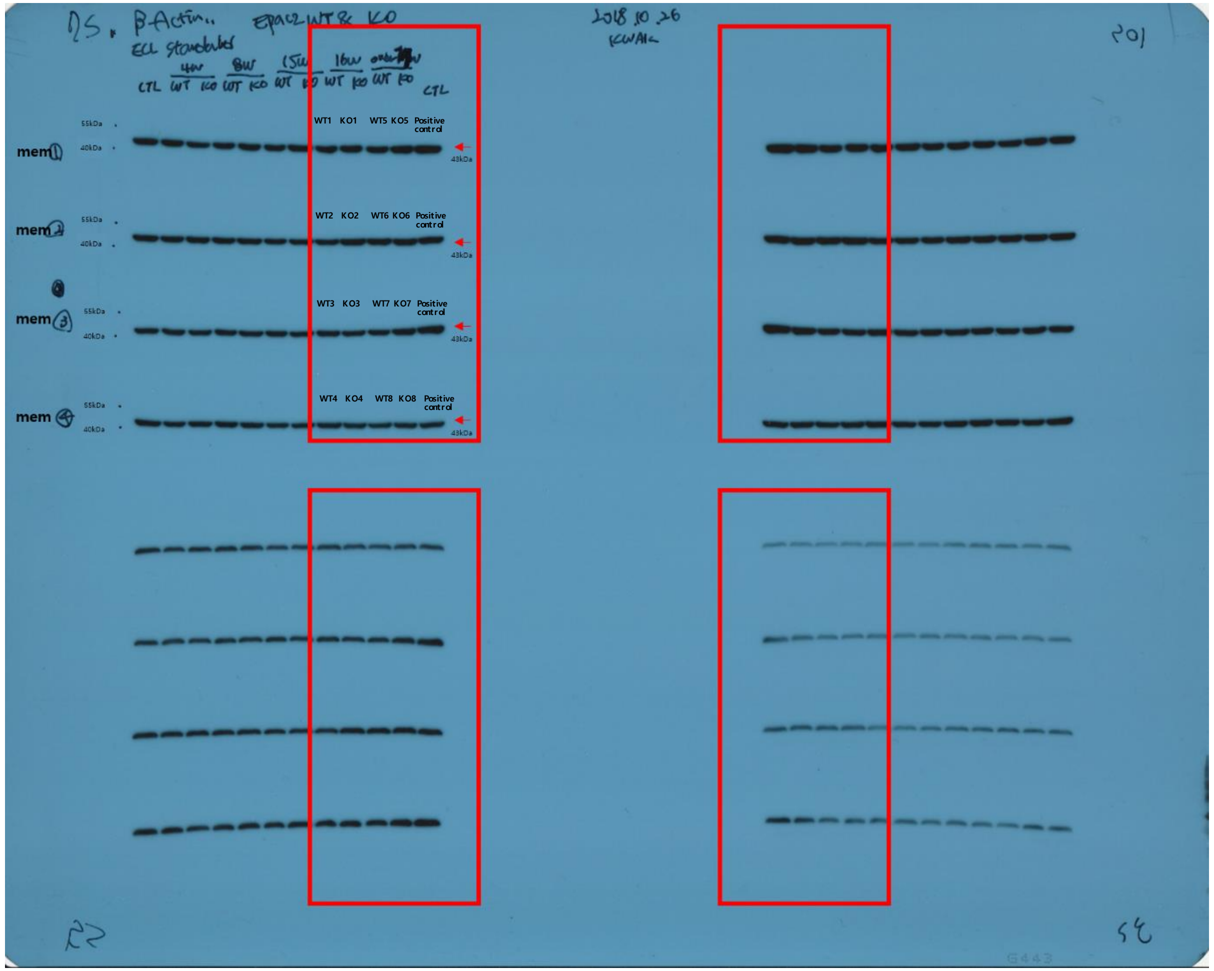


| WT | |
|---|---|
| sample ID | Mouse ID |
| WT1_16wk | 16W WT G40 |
| WT2_16wk | 16W WT G41 |
| WT3_16wk | 16W WT G45 |
| WT4_16wk | 16W WT G57 |
| WT5_over | 17W WT G856 |
| WT6_over | 19W WT G854 |
| WT7_over | 34W WT G1+ G3 |
| WT8_over | 54W WT G1106 + G1108 |

| KO | |
|---|---|
| sample ID | Mouse ID |
| KO1_16wk | 16W KO G868 |
| KO2_16wk | 16W KO G869 |
| KO3_16wk | 16W KO G36 |
| KO4_16wk | 16W KO G60 |
| KO5_over | 17W KO G864 |
| KO6_over | 19W KO G38 + G39 |
| KO7_over | 29W KO G835 |
| KO8_over | 54W KO G1044+ G1115 |

| Positive control | |
|---|---|
| sample ID | Mouse ID |
| positive control 1 | Naïve mouse 1 |
| positive control 2 | Naïve mouse 1 |
| positive control 3 | Naïve mouse 1 |
| positive control 4 | Naïve mouse 1 |
| positive control 5 | Naïve mouse 1 |
| positive control 6 | Naïve mouse 1 |
| positive control 7 | Naïve mouse 1 |
| positive control 8 | Naïve mouse 1 |

**Supple 3**

Trial 2

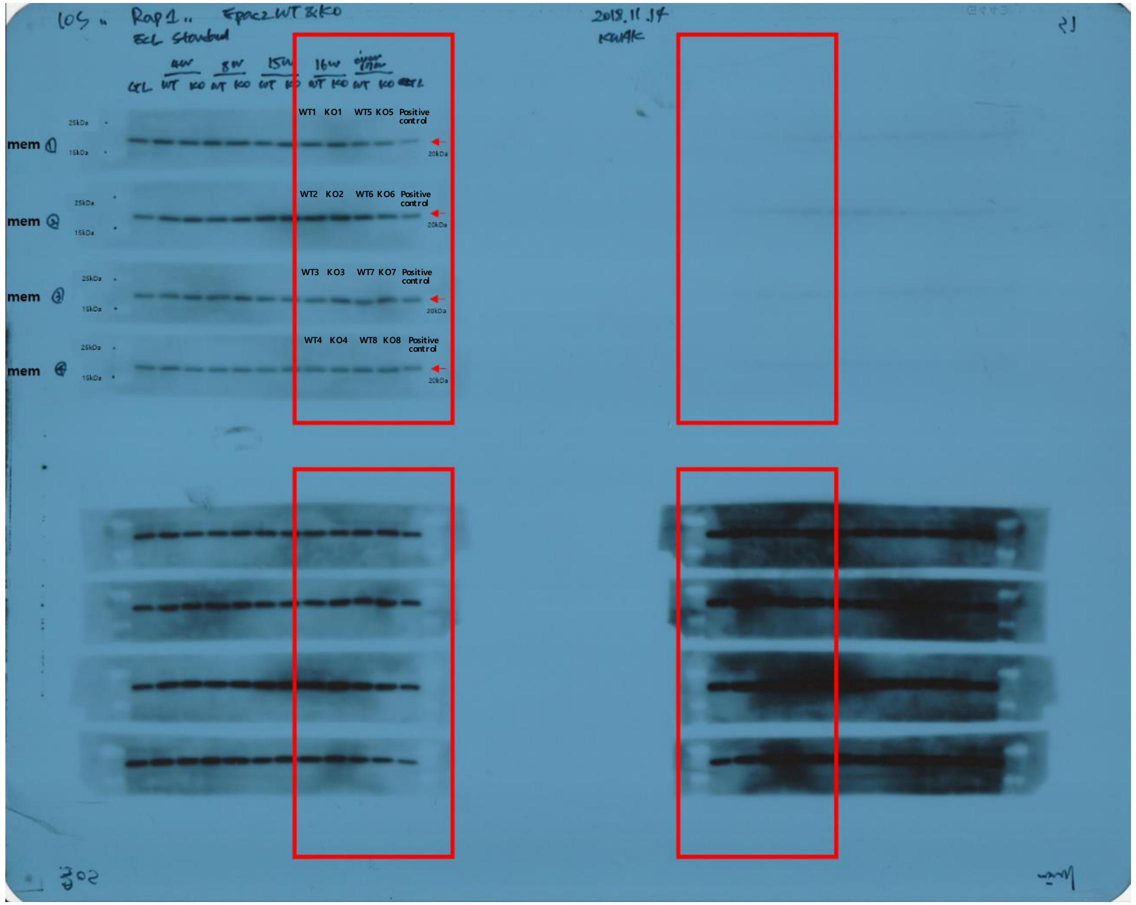


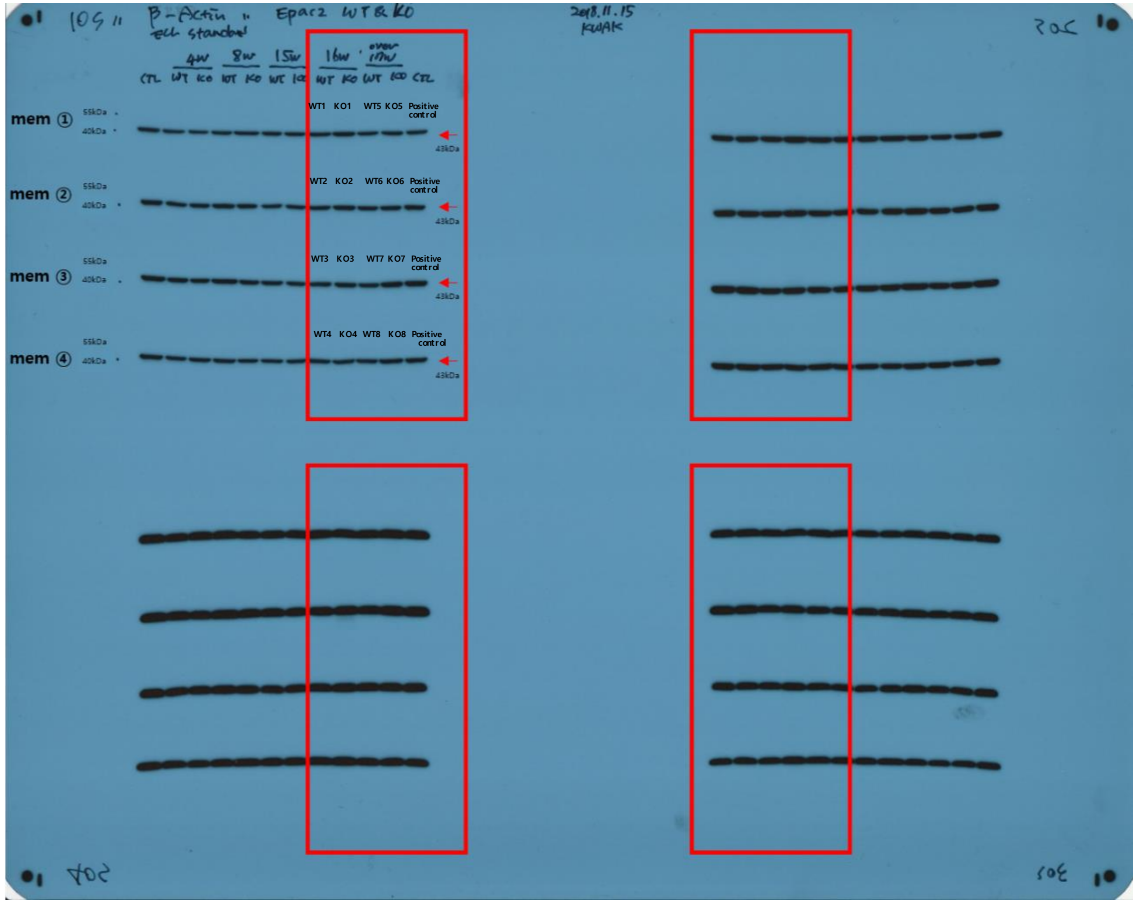


| WT | |
|---|---|
| sample ID | Mouse ID |
| WT1_16wk | 16W WT G40 |
| WT2_16wk | 16W WT G41 |
| WT3_16wk | 16W WT G45 |
| WT4_16wk | 16W WT G57 |
| WT5_over | 17W WT G856 |
| WT6_over | 19W WT G854 |
| WT7_over | 34W WT G1+ G3 |
| WT8_over | 54W WT G1106 + G1108 |

| KO | |
|---|---|
| sample ID | Mouse ID |
| KO1_16wk | 16W KO G868 |
| KO2_16wk | 16W KO G869 |
| KO3_16wk | 16W KO G36 |
| KO4_16wk | 16W KO G60 |
| KO5_over | 17W KO G864 |
| KO6_over | 19W KO G38 + G39 |
| KO7_over | 29W KO G835 |
| KO8_over | 54W KO G1044+ G1115 |

| Positive control | |
|---|---|
| sample ID | Mouse ID |
| positive control 1 | Naïve mouse 1 |
| positive control 2 | Naïve mouse 1 |
| positive control 3 | Naïve mouse 1 |
| positive control 4 | Naïve mouse 1 |
| positive control 5 | Naïve mouse 1 |
| positive control 6 | Naïve mouse 1 |
| positive control 7 | Naïve mouse 1 |
| positive control 8 | Naïve mouse 1 |

# Supple 4

Trial 1

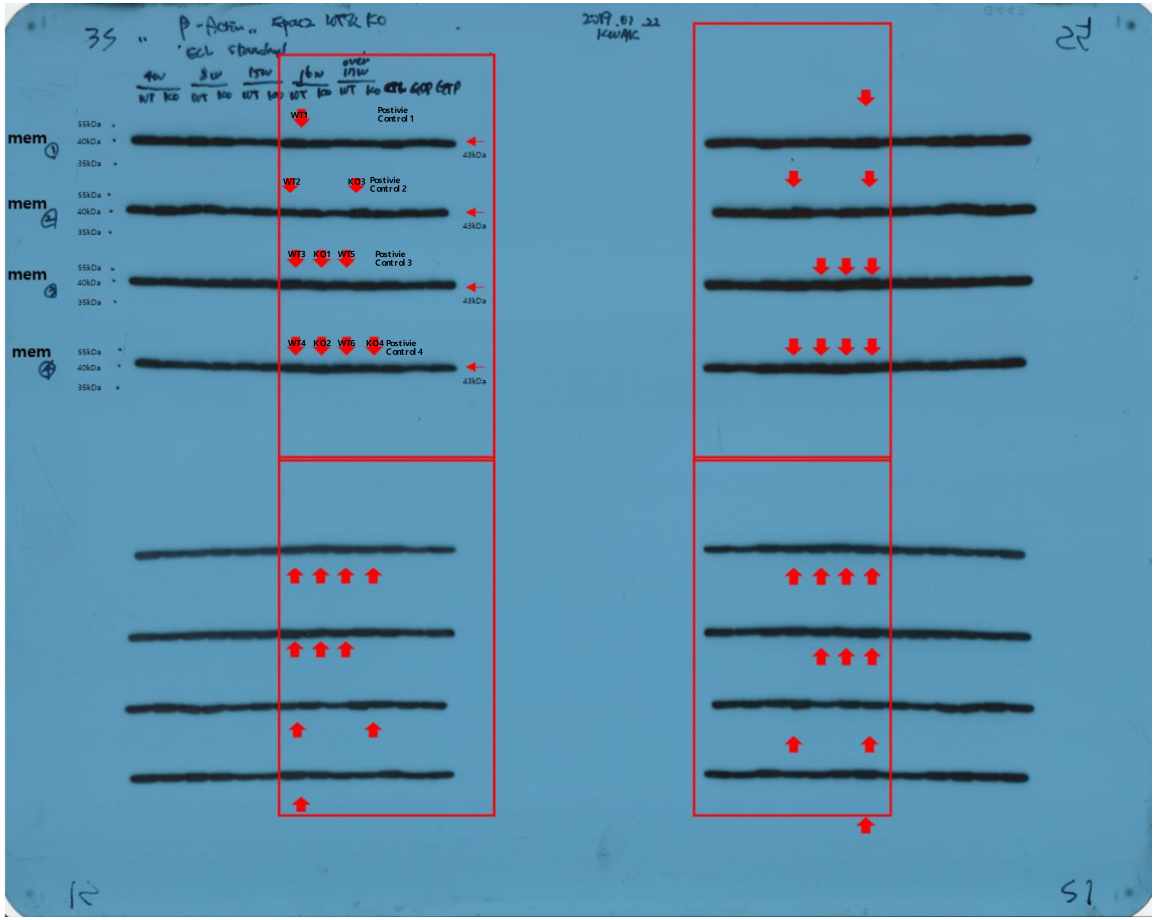


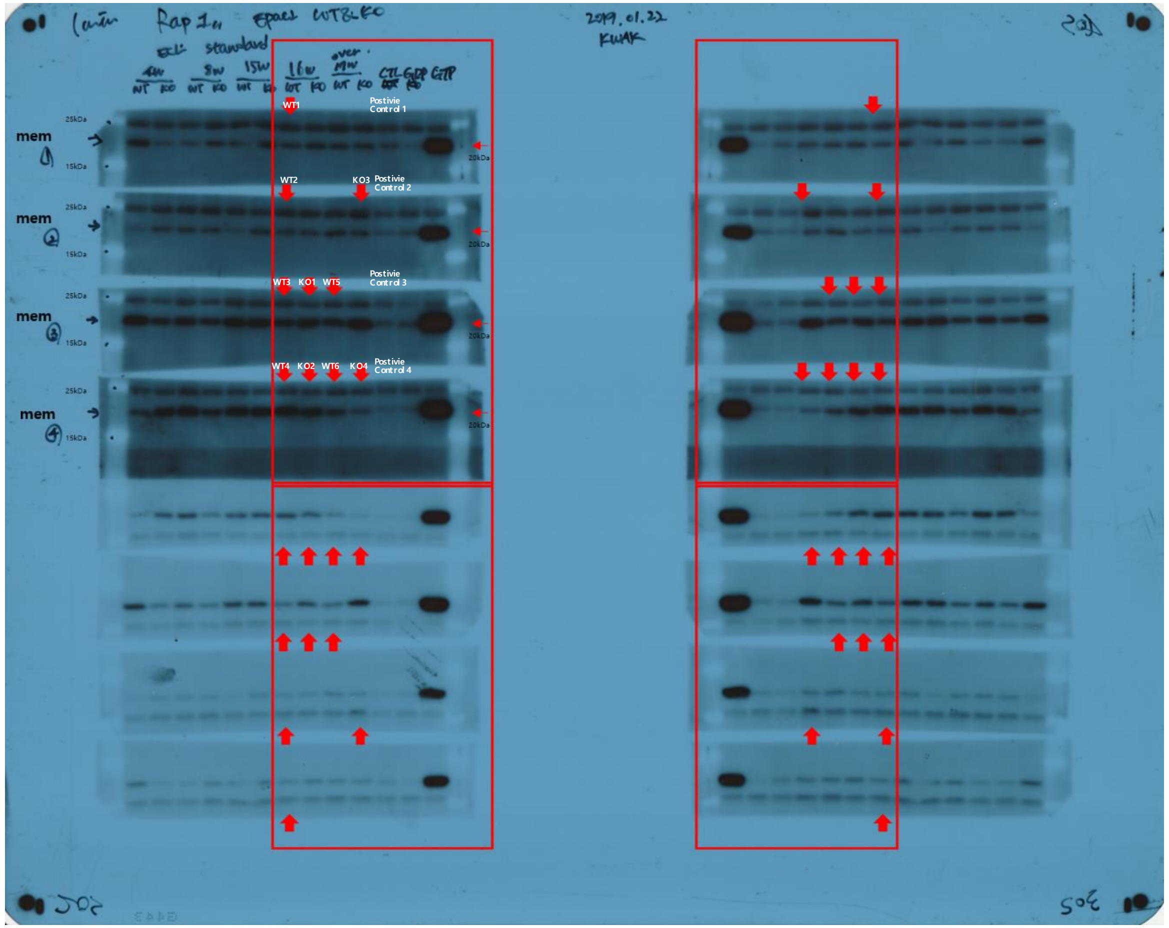


| WT | |
|---|---|
| sample ID | Mouse ID |
| WT1_16wks | 16W WT G40 |
| WT2_16wks | 16W WT G41 |
| WT3_16wks | 16W WT G45 |
| WT4_16wks | 16W WT G57 |
| WT5_over 17ws | 34W WT G1+ G3 |
| WT6_over 17ws | 54W WT G1106 + G1108 |

| KO | |
|---|---|
| sample ID | Mouse ID |
| KO1_16wks | 16W KO G36 |
| KO2_16wks | 16W KO G60 |
| KO3_over 17ws | 19W KO G38 + G39 |
| KO4_over 17ws | 54W KO G1044+ G1115 |

| Positive control | |
|---|---|
| sample ID | Mouse ID |
| positive control 1 | Naïve mouse 1 |
| positive control 2 | Naïve mouse 1 |
| positive control 3 | Naïve mouse 1 |
| positive control 4 | Naïve mouse 1 |

# Supple 4

Trial 2

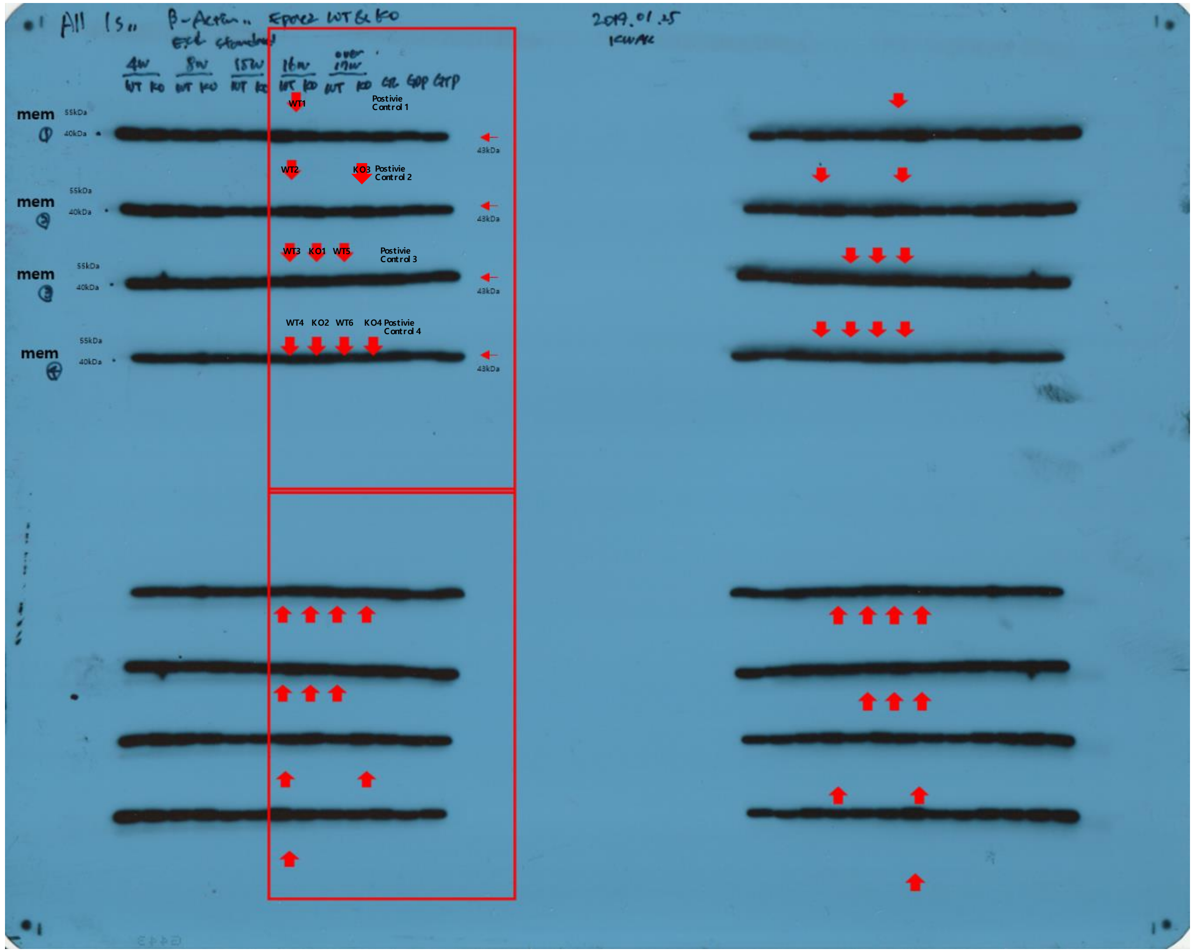


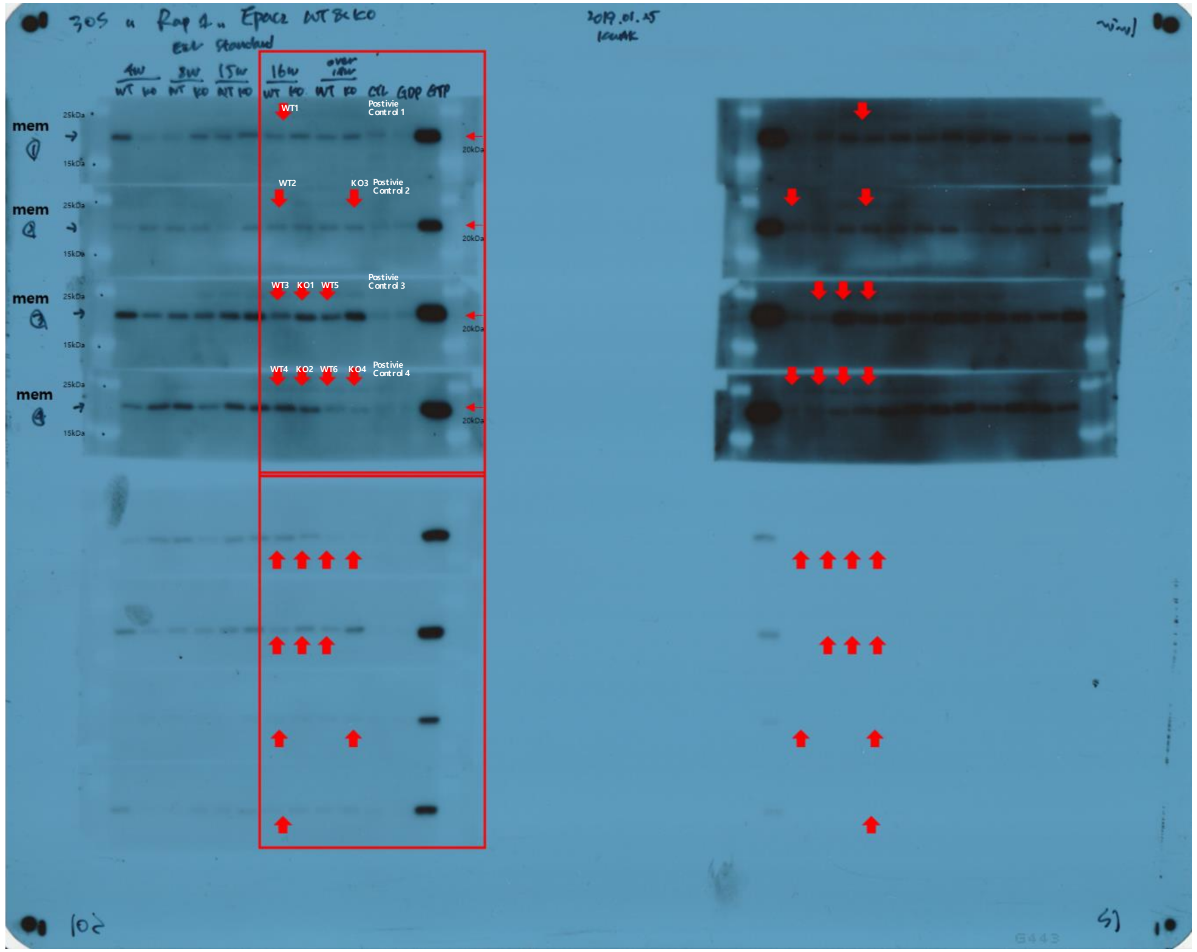


| WT | |
|---|---|
| sample ID | Mouse ID |
| WT1_16wks | 16W WT G40 |
| WT2_16wks | 16W WT G41 |
| WT3_16wks | 16W WT G45 |
| WT4_16wks | 16W WT G57 |
| WT5_over 17ws | 34W WT G1+ G3 |
| WT6_over 17ws | 54W WT G1106 + G1108 |

| KO | |
|---|---|
| sample ID | Mouse ID |
| KO1_16wks | 16W KO G36 |
| KO2_16wks | 16W KO G60 |
| KO3_over 17ws | 19W KO G38 + G39 |
| KO4_over 17ws | 54W KO G1044+ G1115 |

| Positive control | |
|---|---|
| sample ID | Mouse ID |
| positive control 1 | Naïve mouse 1 |
| positive control 2 | Naïve mouse 1 |
| positive control 3 | Naïve mouse 1 |
| positive control 4 | Naïve mouse 1 |

# Supple 5

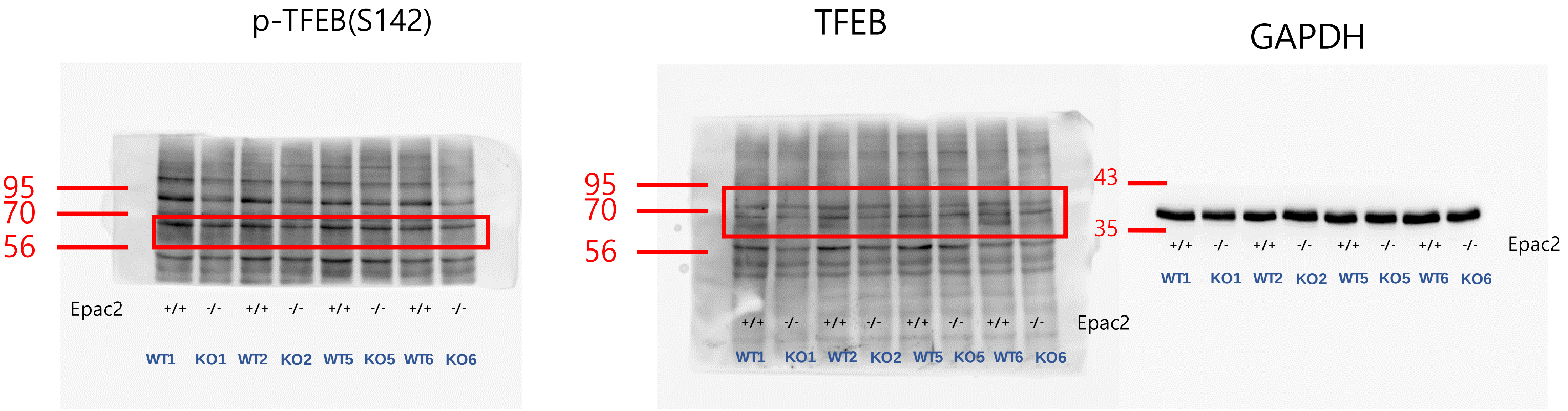


| Sample | Mouse ID | Epac2 $^{+/+}$ | Sample | Mouse ID | Epac2 $^{-/-}$ |
|---|---|---|---|---|---|
| WT1 | 16w WT G40 | 1.296692 | KO1 | 16w KO G868 | 0.6885496 |
| WT2 | 16w WT G41 | 0.8473282 | KO2 | 16w KO G869 | 0.3969466 |
| WT3 | 17w WT G856 | 1.022646 | KO3 | 16w KO G36 | 0.7684478 |
| WT4 | 34w WT G1+G3 | 0.7938932 | KO4 | 17w KO G864 | 0.3104326 |
| WT5 | 54w WT G1106+G1108 | 0.7684478 | KO5 | 96w KO G38+ G835 | 0.3816794 |
| WT6 | 21w WT G859 | 1.272265 | KO6 | 54 w KO G1044+G1115 | 0.7633588 |

## Supple 5

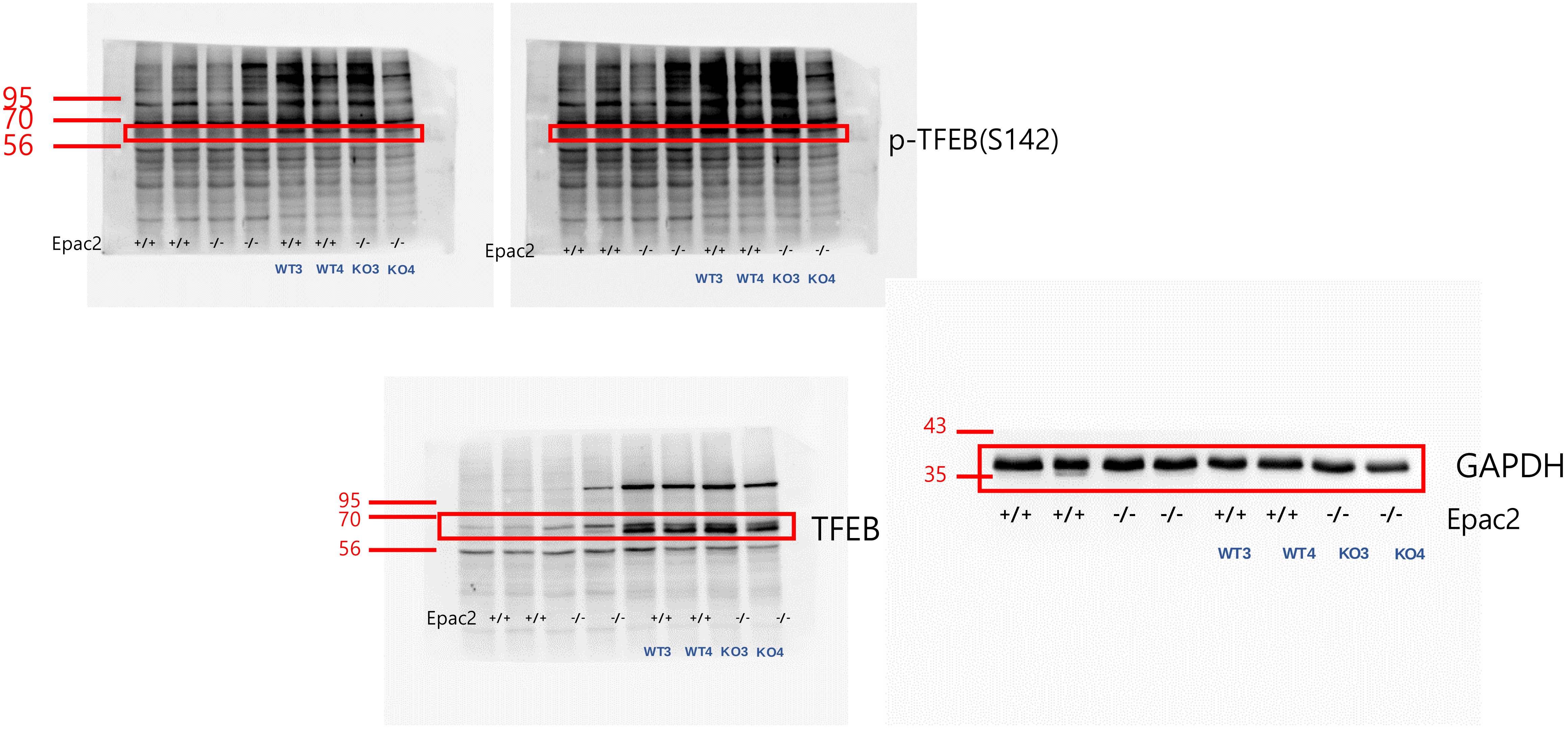
95
70
56
Epac2 +/+ +/+ -/- -/- +/+ +/+ -/- -/-
WT3 WT4 KO3 KO4
Epac2 +/+ +/+ -/- -/- +/+ +/+ -/- -/-
WT3 WT4 KO3 KO4
p-TFEB(S142)
95
70
56
TFEB
Epac2 +/+ +/+ -/- -/- +/+ +/+ -/- -/-
WT3 WT4 KO3 KO4
43
35
GAPDH
+/+ +/+ -/- -/- +/+ +/+ -/- -/- Epac2
WT3 WT4 KO3 KO4
